# Yunnan Baiyao-loaded multifunctional microneedle patches for rapid hemostasis and cutaneous wound healing

**DOI:** 10.1186/s12951-023-01936-w

**Published:** 2023-06-06

**Authors:** Jie Yang, Xiaocheng Wang, Dan Wu, Kexin Yi, Yuanjin Zhao

**Affiliations:** 1 Department of Rheumatology and Immunology, Nanjing Drum Tower Hospital, School of Biological Science and Medical Engineering, Southeast University, Nanjing, 210096 China; 2 Oujiang Laboratory (Zhejiang Lab for Regenerative Medicine, Vision and Brain Health), Wenzhou Institute, University of Chinese Academy of Sciences, Wenzhou, 325001 Zhejiang China

**Keywords:** Microneedle, Hemostasis, Wound healing, Yunnan Baiyao, EGF

## Abstract

Microneedle patches have been extensively employed for wound healing, while the lack of rapid hemostasis efficiency and multiple tissue-repair properties restrict their values in hemorrhagic wound applications. Herein, we propose a Yunnan Baiyao-loaded multifunctional microneedle patch, namely (BY + EGF)@MN, with deep tissue penetration, hemostasis efficiency and regenerative properties for hemorrhagic wound healing. The (BY + EGF)@MNs are designed with a BY-loaded Bletilla striata polysaccharide (BSP) base for rapid hemostasis and epidermal growth factor (EGF)-loaded GelMA tips for subsequent wound healing. As the BSP base can be fastly dissolved and completely release BY in 6 min to promote platelet adhesion and activate coagulation system, while the EGF can achieve a controlled and sustained release behavior in 7 days with the gradual degradation of the GelMA tips, the (BY + EGF)@MNs exhibit strong pro-coagulability and satisfactory hemostatic effect in a rat hepatic hemorrhage wound model. Based on the multifunctional characteristics, we have verified that when applied in rat cutaneous wounds, the proposed MNs can accelerate the wound healing process by enhancing neovascularization, fibroblast density, and collagen deposition. Thus, we believe that such (BY + EGF)@MNs are promising candidates for rapid hemostasis and diverse wound healing applications.

**Graphical Abstract:**

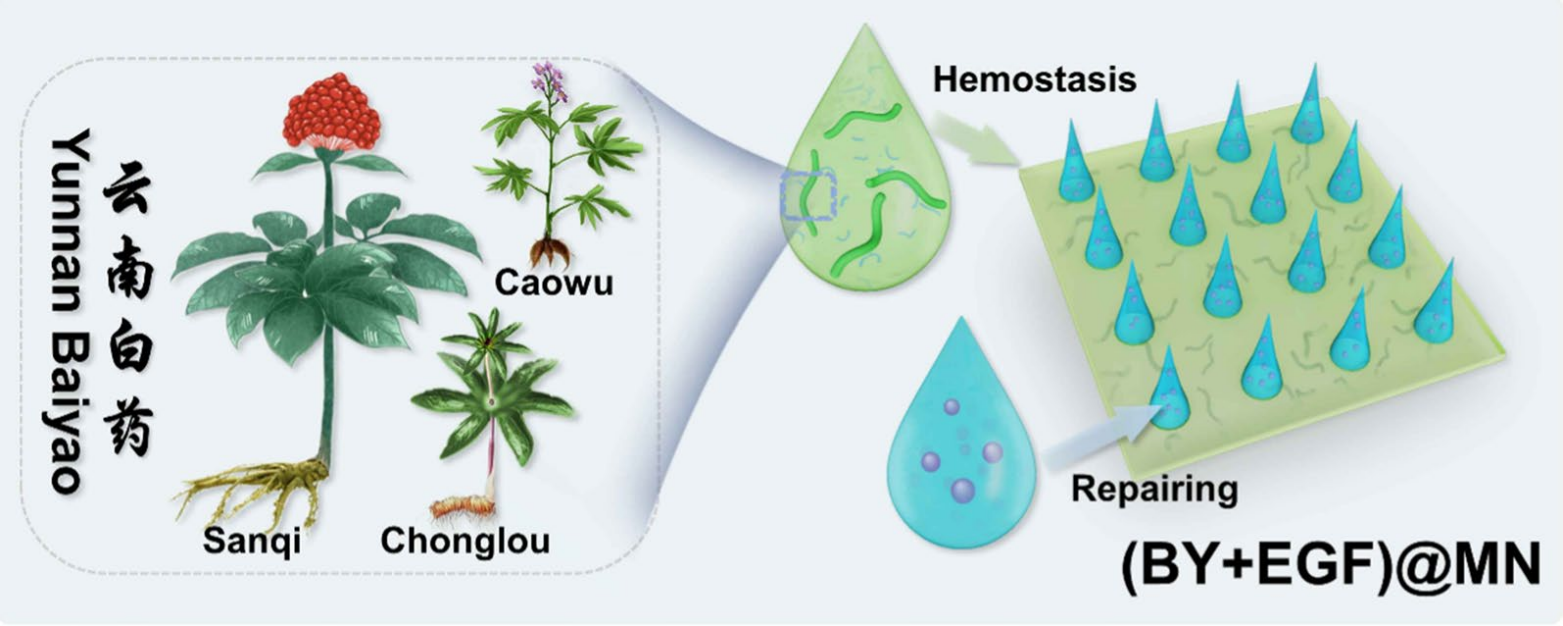

**Supplementary Information:**

The online version contains supplementary material available at 10.1186/s12951-023-01936-w.

## Introduction

Hemorrhagic wounds caused by traffic accidents, natural catastrophes, firearms, and explosions in the battlefield, are serious issues for civilian and military trauma patients worldwide [1–5]. Wound healing typically involves following overlapped phases including hemostasis, anti-inflammation, proliferation, and remodeling, which should be tightly coordinated to efficiently restore the tissue integrity [6–11]. Uncontrolled hemorrhage is the leading cause of more than 50% traumatic death worldwide [12]. Although various current hemostatic agents present effective hemostasis ability for bleeding wounds on the tissue surface, such as injectable glues [13], hemostatic bandages [14], and procoagulant powders [15], they are difficult to reach deep tissues and usually prone to fall off from wound surfaces. By contrast, microneedles (MNs) have emerged as effective transdermal delivery systems, which can overcome the resistance of the stratum corneum and penetrate the skin tissues [16, 17]. Owing to the painless and minimally invasive properties, MNs have been widely employed to deliver a variety of therapeutic agents for wound healing, including nanoparticles [18], chemical drugs [19], growth factors [20], nucleic acids [21], etc. However, most MNs cannot realize the combination of tissue adhesiveness and fast hemostasis, but also hardly synchronize with all stages of dynamic tissue repairing process [22]. Therefore, it is highly anticipated to develop a multifunctional microneedle patch integrating with proper adhesiveness, high hemostasis efficiency, controllable drug release ability and tissue regenerative properties for hemorrhagic wound healing.

**Fig. 1:**
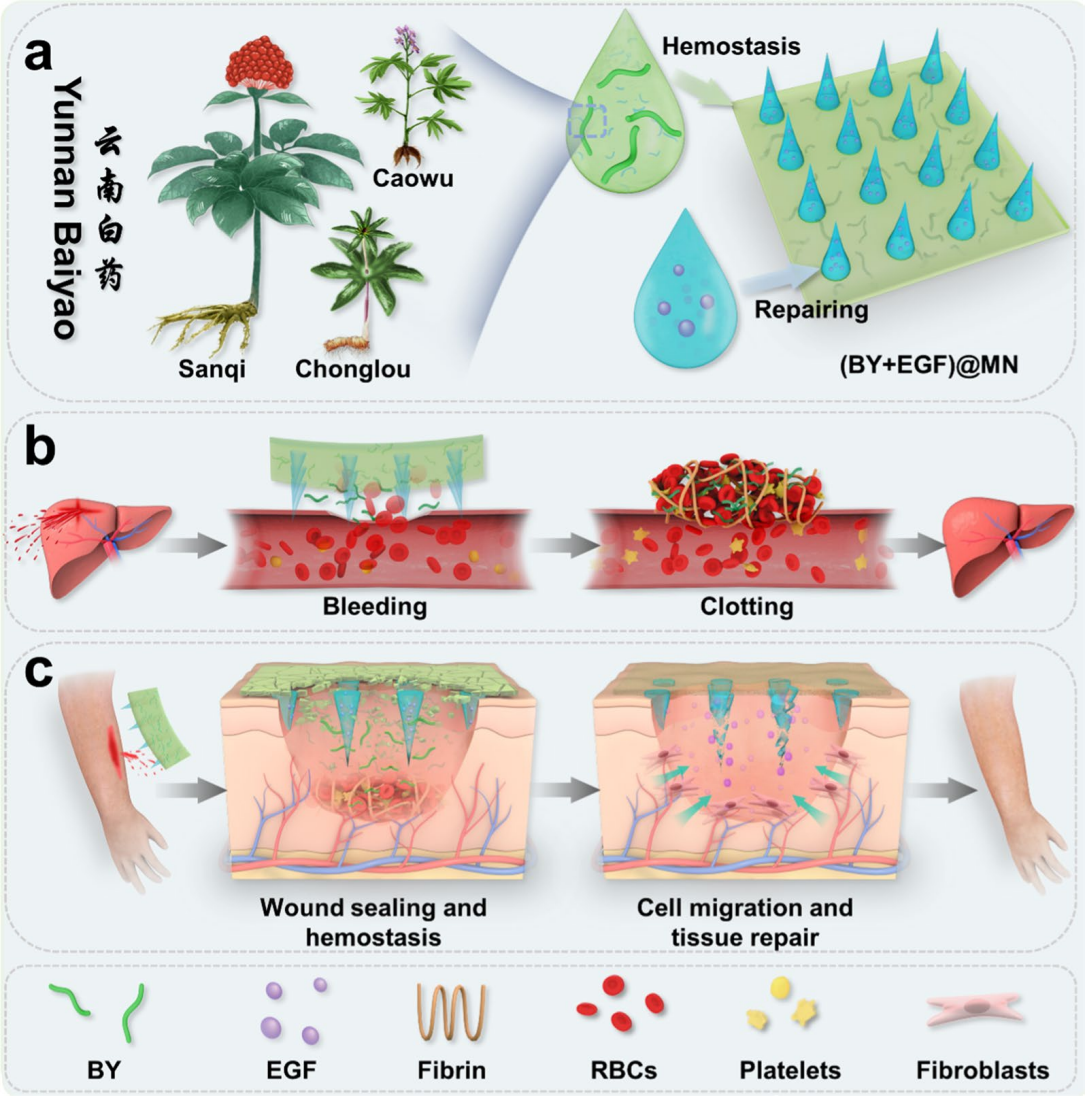
The schematic diagram of Yunnan Baiyao-loaded multifunctional microneedle patches ((BY + EGF)@MNs) for hepatic hemostasis and cutaneous wound repair. **a** Design of (BY + EGF)@MNs with divisional structures. **b** The (BY + EGF)@MNs could exert strong pro-coagulant capacity in a rat hepatic hemorrhage wound model. **c** When applied to cutaneous wounds, the (BY + EGF)@MNs can accelerate the wound healing process by enhancing neovascularization, fibroblast density, and collagen deposition

Herein, we propose a Yunnan Baiyao-loaded multifunctional microneedle patch with the desirable features for hemorrhagic wound healing, as indicated in Fig. 1. Yunnan Baiyao (BY) is known as a traditional Chinese herbal medicinal formula for its hemostatic effect and permitted by the China Food and Drug Administration [23]. The sophisticated herb composition in BY includes Panax notoginseng (Sanqi), Rhizoma Paridis (Chonglou), Radix Aconiti Kusnezoffii (Caowu), Borneolum Synthcticum (Shexiang), and Forest Musk [24]. These components have been previously demonstrated effective to enhance hemostasis, reduce inflammation, and increase antioxidant activity for wound healing [25]. However, the current BY-laden hydrogel patches mainly focus on the hemostatic effects at the early stage of bleeding wounds, while little attention has been paid to the subsequent wound healing after hemostasis. Given the divisional structures [26] and superior drug delivery capacity of MN patches, it is speculated that the stepwise treatment of hemorrhagic wounds could be realized by using a novel BY-laden MN patch with a BY-laden MN base for instantaneous hemostasis at the early stage and EGF-laden MN tips for promoting wound healing at the late stage.

In this manuscript, we present the BY-loaded multifunctional MN patches with divisional structures for hemorrhagic wound healing (Fig. 1). The multifunctional MN patches, namely (BY + EGF)@MNs, were fabricated via a two-step template replication method, which imparted the MNs with the BY-loaded Bletilla polysaccharide (BSP) bases for rapid hemostasis and epidermal growth factor (EGF)-loaded GelMA tips for subsequent wound healing. It was demonstrated that the BSP base could be fast dissolved and completely release BY in 6 min to promote platelet adhesion and activate coagulation system, while the EGF showed a controlled and sustained release behavior in 7 days with the gradual degradation of the GelMA tips to accelerate wound healing process. Thus, compared with clinically-used gauzes, the (BY + EGF)@MNs showed stronger pro-coagulability in vitro and better hemostatic effect in a rat hepatic hemorrhage wound model. Furthermore, when we applied the (BY + EGF)@MNs in the rat cutaneous wounds, they could obviously accelerate the tissue remolding process by enhancing neovascularization, fibroblast density, and collagen deposition. All these motivative results indicate the practical values of the (BY + EGF)@MNs for rapid hemostasis and cutaneous wound healing.

## Results and discussion

### Characterization of the (BY + EGF)@MNs

**Fig. 2:**
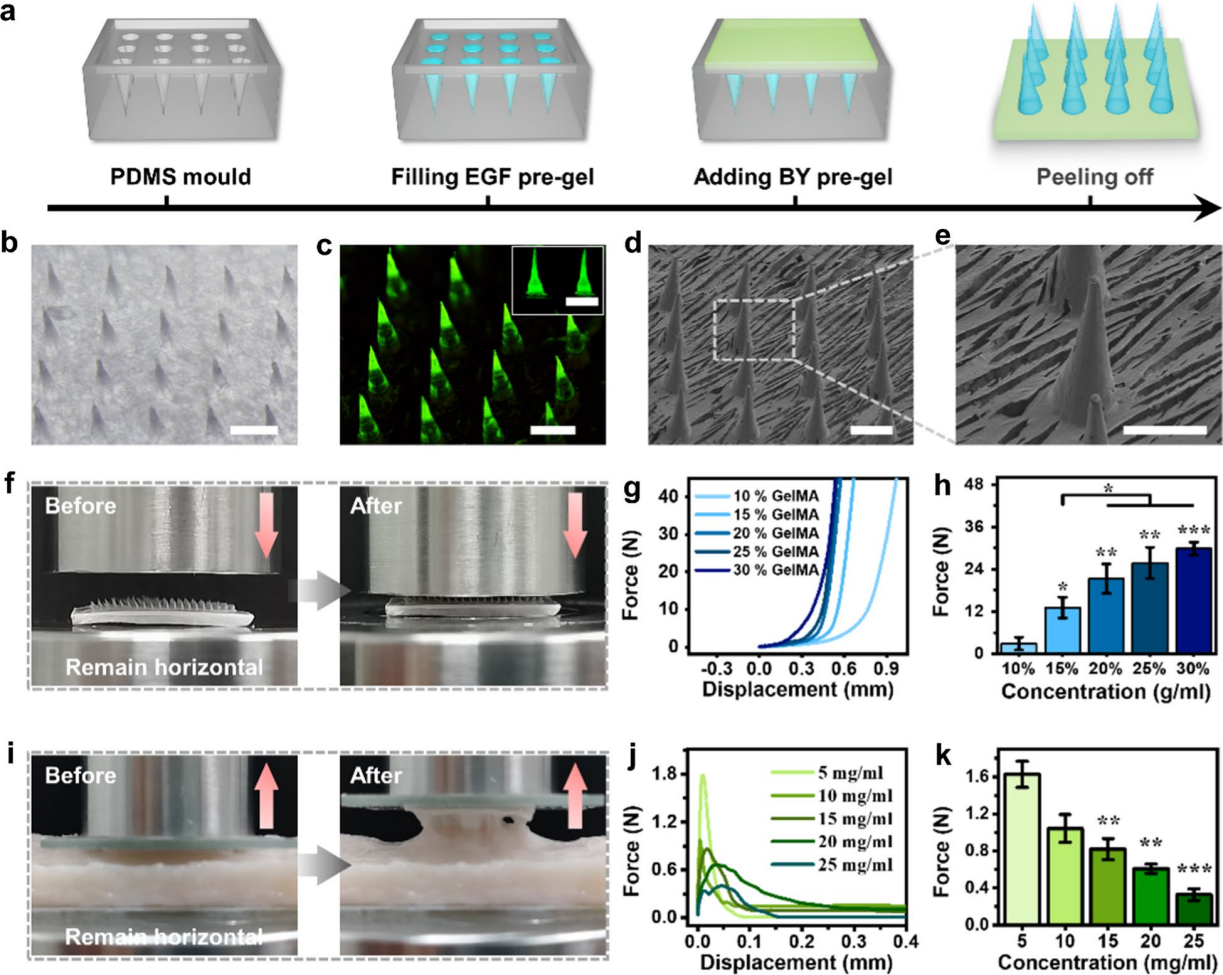
Morphological and mechanical characterization of the (BY + EGF)@MNs. **a **The fabricated process diagram of the (BY + EGF)@MNs. **b** Optical bright-filed and **c** corresponding fluorescent images of the (BY + EGF)@MNs. **d**, **e** SEM images of the MN tips of the (BY + EGF)@MNs at different magnifications. **f** The mechanical compression test of MN tips. **g** The force-displacement curves of MN tips with gradient GelMA. **h** The compression force when the sensor moved 500 μm (n = 4). **i** The mechanical tensile test of MN bases. **j** The force-displacement curves of the MN bases with gradient carbomer contents. **k** The maximum adhesion force between pig skin and MN bases with gradient carbomer contents (n = 3). Scale bars are 500 μm in (**b**–**e**)

Firstly, GelMA was synthesized from gelatin, and its characteristic peaks of the double bond could be observed at 5.32 and 5.55 ppm by NMR spectroscopy, which indicated the degree of substitution to be approximately 90 ± 5% (Additional file 1: Fig. S1). Typically, the (BY + EGF)@MNs was fabricated by a micro-molding approach. As shown in Fig. 2a, EGF was uniformly distributed in the GelMA solution to obtain the EGF pre-gels, and BY was uniformly distributed in the BSP solution with carbomer as a thickener to obtain the BY pre-gels. For the preparation of EGF-loaded MN tips, the polydimethylsiloxane (PDMS) moulds were firstly filled with the EGF pre-gels using a vacuum pump and then polymerized under UV irradiation. For the preparation of BY-loaded MN bases, the excess EGF pre-gels were removed, and the BY pre-gels were filled in the MN moulds and then lyophilized at − 80 ℃. The (BY + EGF)@MNs could be peeled off from moulds, and the entire MN tips were arranged neatly in an 20 × 20 array (Additional file 1: Fig. S2a). Each MN tip possessed a conical shape with a height about 750 μm and a bottom diameter about 210 μm, and the distance between the two adjacent tips is about 650 μm (Fig. 2b, c). Moreover, the microstructure of (BY + EGF)@MNs were photographed by scanning electron microscopy (SEM), revealing a solid structure of the MN tips from inside to outside (Fig. 2d,e and Additional file 1: Fig. S2b,c), which could endow the MNs with strong puncture strength for further in vivo application.

In addition, we verified the mechanical strength of the (BY + EGF)@MNs using the universal mechanical testing machine. As shown in Fig. 2f, a MN patch was placed on a platform with its MN tips pointing to the descending sensor, which recorded the force-displacement curves. Figure 2g showed that the MN tips with a higher GelMA concentration possessed a stronger force at the same displacement. The maximum compression forces at the MN tips (in 500 μm, Fig. 2h) with 10%, 15%, 20%, 25%, 30% GelMA were 2.88 ± 1.82 N, 13.13 ± 2.95 N, 21.33 ± 4.18 N, 25.78 ± 4.45 N, 29.89 ± 1.82 N, respectively, indicating an increased mechanical compression capacity with increasing GelMA concentrations. Besides, the successful penetration with (BY + EGF)@MNs into skin and liver of rats was further demonstrated by the cross-sectional hematoxylin and eosin (H&E) staining photographs (Additional file 1: Fig. S3). It was found that the MN tips could penetrate the skin epidermis layer and the liver capsules, ensuring sufficient penetration depth for EGF delivery at the puncture sites. In addition, the adhesion ability of the MN bases to wounds was evaluated by a tensile test. As shown in Fig. 2i, the MN bases with gradient carbomer concentrations were attached to the upside pressure sensor, which gradually moved away from the pig skin tissues on the horizontal stage. The force-displacement curves (Fig. 2j) and the maximum adhesion force of MN bases (Fig. 2k) were recorded, indicating a decreased adhesion with increasing carbomer concentrations. All above results demonstrated the satisfactory tissue penetration and adhesion ability of the (BY + EGF)@MNs, which could fulfill multifunctional requirements by delivery different drugs in both the MN bases and tips in practical applications.

### In vitro biodegradation and drug delivery

**Fig. 3:**
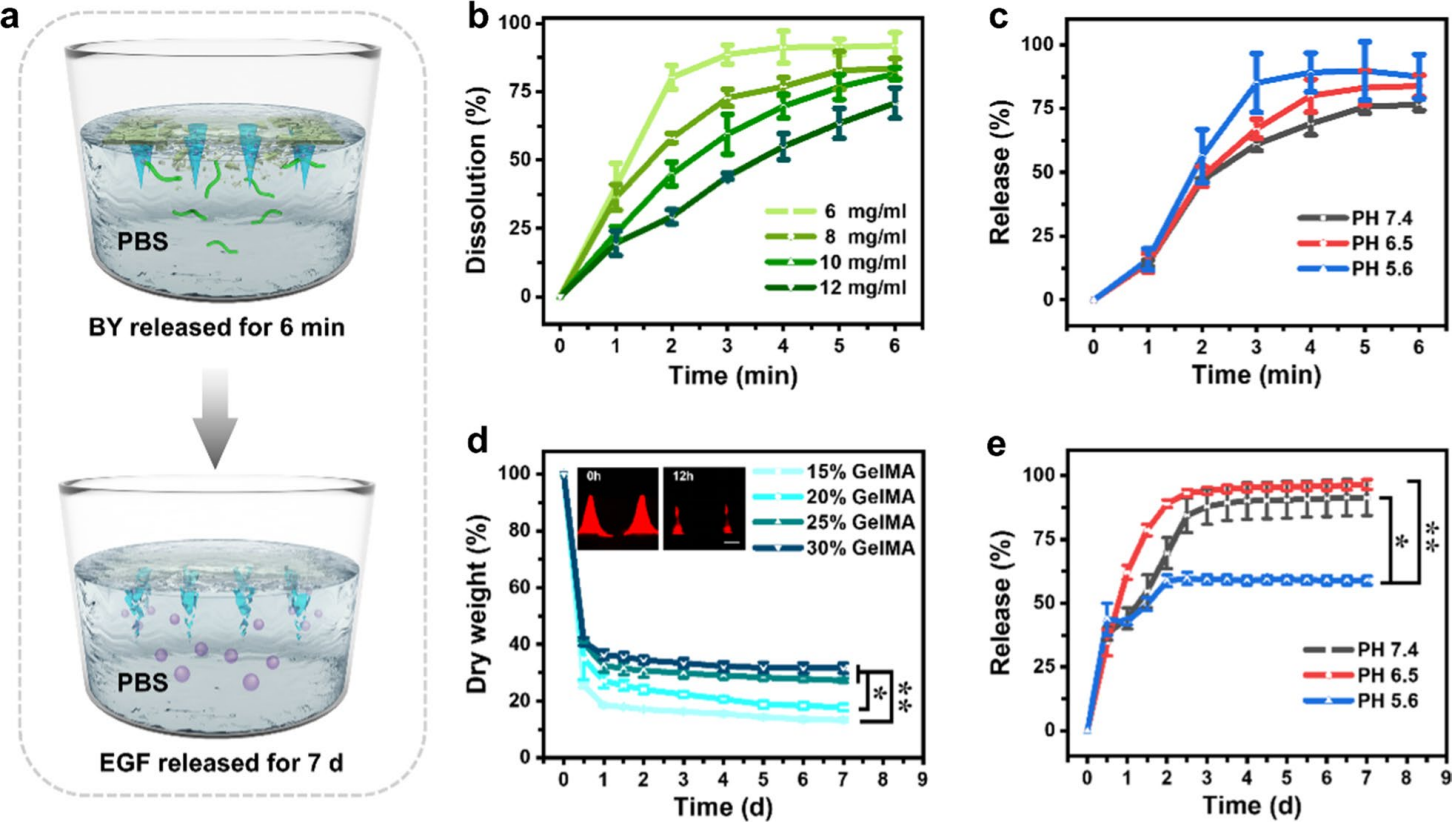
Biodegradation and drug release of the (BY + EGF)@MNs. **a** Schematic of drug release of (BY + EGF)@MNs. **b** The dissolution curves of MN bases with the gradient carbomer contents (n = 3). **c** Release curves of the BY from (BY + EGF)@MNs at different pH (n = 3). **d** Degradation of (BY + EGF)@MNs with different GelMA concentrations in 2.6 U/ml collagenases (n = 3), and corresponding fluorescence images of degradative MN tips after 0 and 12 h. **e** Release curves of the EGF from (BY + EGF)@MNs at different pH (n = 3). The scale bar is 200 μm in (**d**)

The in vitro biodegradation and drug delivery ability of (BY + EGF)@MNs were subsequently evaluated in varied pH environments (i.e., pH = 5.6, 6.5, and 7.4). As illustrated in Fig. 3a, the MN bases were mainly composed of BSP hydrogels, which could be dissolved rapidly to release BY (within several minutes). In addition, with the presence of collagenase in the body, the GelMA MN tips will be gradually degraded and release EGF (within few days). The BY and BSP concentrations could be detected by measuring their UV spectra using a UV spectrophotometry. BSP had a higher absorbance at 260 ~ 340 nm, while the maximum absorbance of BY was at 260 nm, and the carbomer had no obvious absorbance between 200 and 400 nm (Additional file 1: Fig. S4). As shown in Fig. 3b, the dissolution rate of BSP-carbomer hydrogels decreased from 91.91 ± 4.72% to 70.89 ± 5.55% with increasing carbomer concentrations from 6 to 12 mg/ml within 6 min. In specific, BSP hydrogels with the carbomer content of 6 mg/ml could be dissolved by 88.67 ± 3.50% in 3 min, which greatly benefited the quick release of BY from the MN base to offer efficient blood coagulation instantaneously. To ensure a quick release of BY and a strong adhesion of MN bases, the carbomer concentration of 6 mg/ml was selected as an optimized concentration in following experiments. As pH tends to be 7.35–7.45 during practical bleeding, we examined the dissolvability and BY release in pH 7.4. Our result showed that the MN bases could release 60.96% of BY within 3 min, which could be of great help to promote blood coagulation in bleeding wounds (Fig. 3c). It is noted that the MN bases were not completely dissolved and appeared in a gel-solid mixture form, which was favorable to form adhesive barriers and cover the bleeding sites.

The MN tips were mainly composed of GelMA hydrogels, which were difficult to be completely degraded when the concentration exceeded 20% in 7 days (Fig. 3d). Considering the puncture strength and appropriate releasing speed, the GelMA concentration of 20% was selected as an optimal concentration for preparing the MN tips, which could be degraded over 50% in 12 h (Fig. 3d). As pH tends to be weakly acidic at wound beds, we examined the EGF release from GelMA tips in pH 6.5 and pH 7.4. It could be seen that 62.10% and 44.09% of EGF could be released within 24 h in pH 6.5 and 7.4, respectively (Fig. 3e). Collectively, these results suggested the excellent biodegradation and drug delivery capacity of (BY + EGF)@MNs.

### In vitro pro-coagulant ability and biocompatibility of (BY + EGF)@MNs

**Fig. 4:**
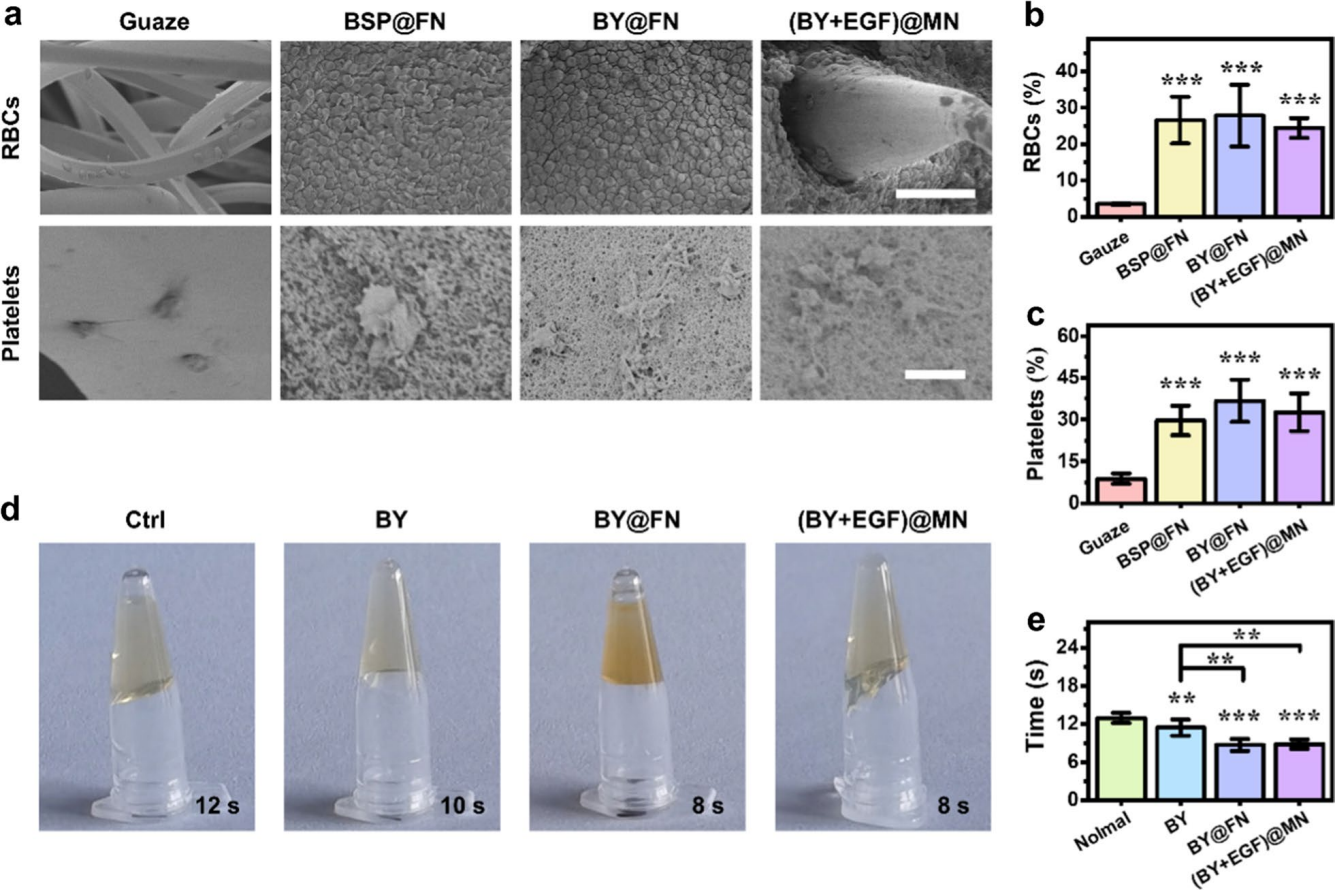
In vitro pro-coagulability of the (BY + EGF)@MNs. **a** SEM photographs displaying the adhesion of RBCs and platelets. Scale bars are 50 μm in (**a,** top), and 3 μm in (**a**, bottom). **b**, **c** Quantification data of adhered RBCs (**b**) and platelets (**c**) in various groups (n = 5). **d** Visualization images of prothrombin times (PT). **e** Quantification data of PT (n = 5)

Subsequently, we verified the hemostatic function of (BY + EGF)@MNs via typical RBC and platelet adhesion assays. SEM images showed that thecell numbers of RBCs and platelets on the BSP@FN (BSP flat patches), BY@FN (BY-loading BSP flat patches), and (BY + EGF)@MNs (MN patches with a BY-loading BSP base and EGF-loading GelMA tips) was higher than that on the gauze (Fig. 4a). Quantitatively, the adhesion rates to RBCs and platelets in the (BY + EGF)@MN group were 24.42 ± 2.69% and 32.55 ± 6.81%, respectively, which was comparable to the BSP@FN (26.58 ± 6.42%, 29.61 ± 5.28%) and BY@FN (27.82 ± 8.48%, 36.67 ± 7.60%) groups, but remarkably higher than the Gauze group (3.47 ± 0.18%, 8.82 ± 1.89%, Fig. 4b, c), suggesting that the BSP MN bases were favorable for the adhesion of RBCs and platelets.

In addition, BY has been proved to facilitate platelet activation, and thereby accelerate thrombosis and hemostasis [27]. As observed in Fig. 4a, the platelets in the BY@FN and (BY + EGF)@MNs underwent morphological deformation and exhibited a large number of pseudopodia, which further facilitated the platelet adhesion to the materials. The change from a smooth bulging appearance to a polygonal or polypseudopodia shape indicated that the activation of the platelets whose cytoskeleton protein underwent contraction when exposed to the samples. Interestingly, plenty of RBCs and platelets attached the MN bases while few cells were adherent to the MN tips as observed in the (BY + EGF)@MN group (Additional file 1: Fig. S5a), indicating the MN bases with stronger adhesion for aggregating more RBCs and platelets. For detecting platelet activation, the expression of P-selectin (CD 62p, a recognized activated indicator [28]) was detected and our result revealed a higher expression of P-selectin on the platelet surface in the BY@FN and (BY + EGF)@MN groups than the BSP@FN and control groups (Additional file 1: Fig. S5b), which confirmed that the BY released from MN bases had a significant promoting effect on platelet activation. As a shorter coagulation time reveals a stronger procoagulant ability, we evaluated the procoagulant ability of (BY + EGF)@MNs by measuring the prothrombin time (PT) and activated partial thromboplastin time (APTT). We found that both BY@FN and (BY + EGF)@MN groups had shorter PTs as compared with the Control and BY groups (Fig. 4d, e), indicating that the BSP base could significantly reduce the exogenous coagulation time. By contrast, there were no significant differences among the APTTs (a normal range of 29–33 s, Additional file 1: Fig. S6) in all groups, suggesting the negligible effects of BY and BSP on the endogenous coagulation pathway. Collectively, these results demonstrated that the synergistic pro-coagulant ability of BSP and BY endowed the (BY + EGF)@MNs with strong hemostatic effects.

Prior to animal experiments, the hemocompatibility and cytocompatibility of (BY + EGF)@MNs were verified to ensure their biosafety. In the hemocompatibility test, no obvious damage to RBCs could be found in the EGF solutions with concentrations less than 5 mg/mL (Additional file 1: Fig. S7a). Notably, a higher concentration of the BY solution was potentially toxic to RBCs, and the RBCs reduced to 85.59% in the BY solution with a concentration of 0.4 mg/ml (Additional file 1: Fig. S7b). After determining the concentration of each component, we evaluated the hemolysis rate of the whole (BY + EGF)@MNs. It was found that when (BY + EGF)@MNs concentration was kept in 4–6 mg/ml, the hemolysis rate was as low as 4.13–5.96%. (Additional file 1: Fig. S7c). For cytocompatible test, we cultured NIH3T3 cells with the leaching solution of (BY + EGF)@MNs. As displayed in Additional file 1: Fig. S8a, the OD value at 450 nm detected by CCK-8 assay revealed that cell proliferation could be significantly enhanced by the (BY + EGF)@MNs than control group. Compared to the control group, live/dead cell staining further confirmed an increased number of living cells in (BY + EGF)@MN group (Additional file 1: Fig. S8b), indicating the good cytocompatibility of (BY + EGF)@MNs. In addition, a scratch wound-healing assay showed that the migration of NIH3T3 cells was significantly accelerated when treated with EGF solutions with concentrations above 40 ng/ml (Additional file 1: Fig. S9). All above results suggested the regenerative potential of (BY + EGF)@MNs to promote cell proliferation and migration in further wound healing applications.

### In vivo hemostatic function of (BY + EGF)@MNs

**Fig. 5:**
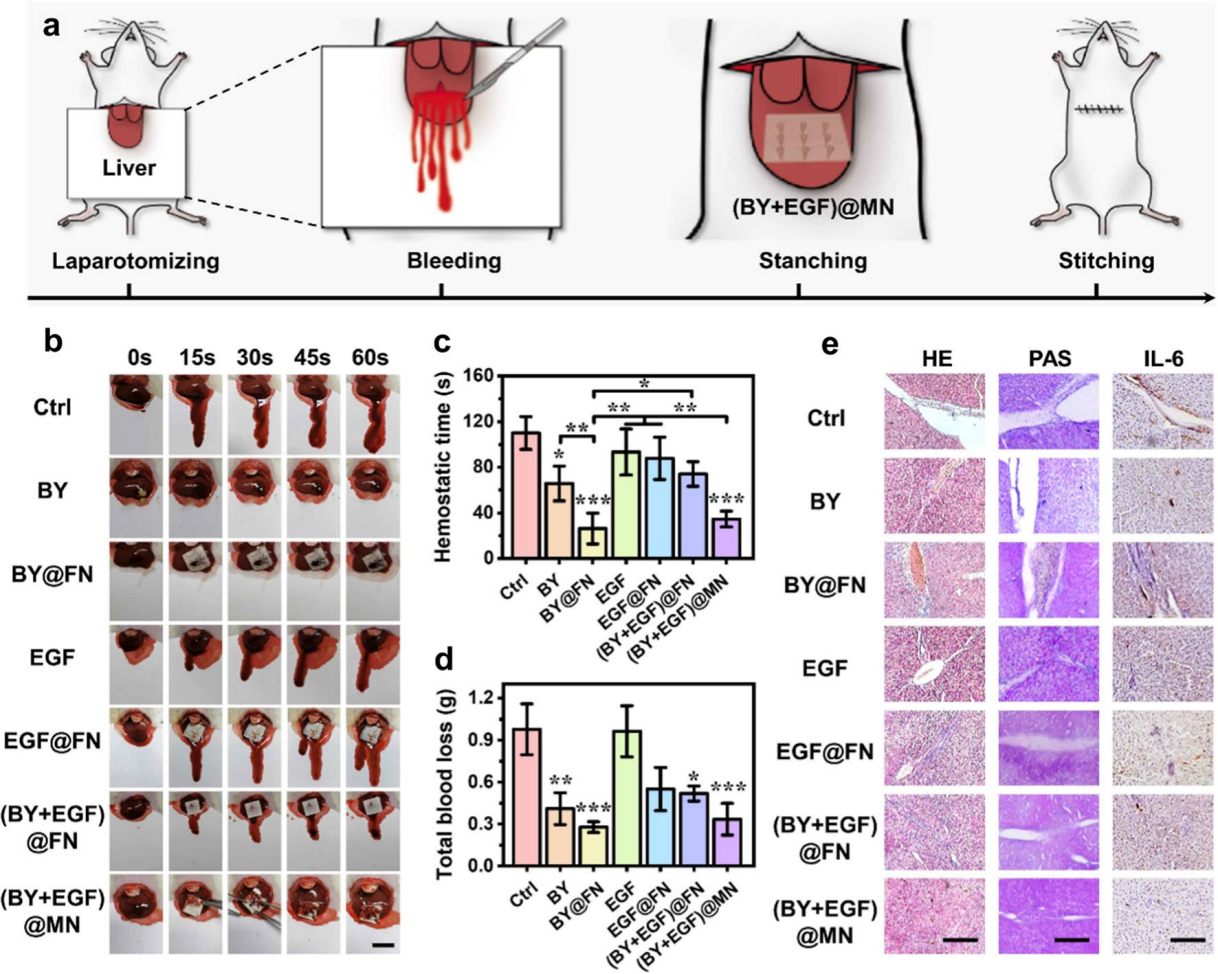
In vivo hemostatic and regenerative capacities of (BY + EGF)@MNs. **a** Schematic diagram showing the hemostatic process using (BY + EGF)@MNs at rat liver wounds. **b** Hemostatic photographs in different groups. **c** Hemostatic time and **d** total blood loss in different groups (n = 3). **e** H&E staining, PAS staining, and immunohistochemistry staining of IL-6 on day 28. Scale bars are 2 cm in (**b**), 400 μm in (**e**, left), 200 μm in (**e**, middle), and 400 μm in (**e**, right)

In vivo hemostatic capacity of (BY + EGF)@MNs was explored using a typical rat hepatic hemorrhage wound model, as showed in Fig. 5a. The hemorrhaging was induced by creating a perforation wound on the liver, and then treated with (BY + EGF)@MNs (i.e.,MN patches with a BY-loading BSP base and EGF-loading GelMA tips) in comparison to other control samples including BY (BY powders), BY@FN (BY-loading BSP flat patches, also called MN base), EGF (EGF solution), EGF@FN (EGF-loading GelMA flat patches), (BY + EGF)@FN (BY-loading BSP and EGF-loading GelMA superimposed flat patches) (Fig. 5b). Less bloodstain was directly observed on the filter paper in the BY-containing patch groups than other groups. Quantitatively, the hemostatic time and the total blood loss of the BY@FN (26.33 ± 11.09 s, 0.27 ± 0.03 g) and (BY + EGF)@MN (34.67 ± 5.73 s, 0.33 ± 0.09 g) was shorter and less than that of the BY (65.67 ± 12.50 s, 0.41 ± 0.09 g), EGF (93.33 ± 16.52 s, 0.96 ± 0.15 g), EGF@FN (87.67 ± 15.17 s, 0.55 ± 0.13 g), and (BY + EGF)@FN (74.00 ± 8.83 s, 0.51 ± 0.04 g) groups (Fig. 5c, d). Notably, the hemostasis effects of the groups containing BY were superior to other groups without BY. The hemostasis effects of BY, BY@FN, (BY + EGF)@MN were better than that of (BY + EGF)@FN which was fabricated from stacking a BY-loading BSP patch (BY@FN) and a EGF-loading GelMA patch (EGF@FN). During the in vivo experiment, (BY + EGF)@MN was applied with the EGF-loading GelMA tips inserted into wound beds and BY-loading BSP bases covering the wounds. Likewise, for the (BY + EGF)@FN group, the EGF@FN side of (BY + EGF)@FN directly contacted the hemorrhagic wound, while the BY@FN side was away from the wounds. Therefore, the hemostasis effect of (BY + EGF)@FN was inferior to BY@FN probably due to the indirect contact of the BY-containing patch to the hemorrhagic wounds, weakening the hemostatic effect of BY@FN. Because BY powders could directly destroy a large number of RBCs when applied in hemorrhagic wounds. On the whole, BY@FN and (BY + EGF)@FN showed excellent therapeutic effect in vivo hemostasis experiments, which was attributed to the strong blood agglutination and adhesion of MN base. We notified that the BSP-based MN base could be quickly dissolved while encountering rat’s blood, leading to obvious stickiness with the tweezer that we used to transfer the (BY + EGF)@MNs to the wounds (Fig. 5b). Therefore, we used another tweezer to separate the prior tweezer and MN base. It should be pointed out that there was no artificial force to press the wounds during the whole hemostatic process.

Such a small liver wound rarely affected the overall liver function in rats, and the rat livers were harvested for evaluating the liver regeneration after 28 days. The healthy ALT/AST levels could reflect the normal hepatocyte function in all rats (Additional file 1: Fig. S10). More importantly, the H&E staining revealed a complete wound closure in the live tissues from the (BY + EGF)@MN group (Fig. 5e), suggesting the in vivo tissue regenerative capacity of (BY + EGF)@MNs. Hepatic glycogen plays a crucial role in stabilizing cellular blood glucose levels [29]. The Periodic Acid Schiff (PAS) staining revealed a widely-distributed hepatic glycogen in the (BY + EGF)@MN group (Fig. 5e). Inflammation response of (BY + EGF)@MNs was evaluated by IL-6 immunohistochemical staining, and the quantitative results (Additional file 1: Fig. S11) indicated the inflammation response in all groups containing BY were significantly reduced as compared to other groups without BY, indicating the good anti-inflammatory effect of BY. In addition, the anti-inflammatory ability of (BY + EGF)@FNs was inferior to BY@FNs and (BY + EGF)@FNs probably due to the indirect contact of the BY-containing patch to the hemorrhagic wounds when treated with (BY + EGF)@FNs. Taken together, all above results demonstrated the significant in vivo hemostatic and regenerative capacities of (BY + EGF)@MNs.

### In vivo cutaneous wound healing of (BY + EGF)@MNs

**Fig. 6:**
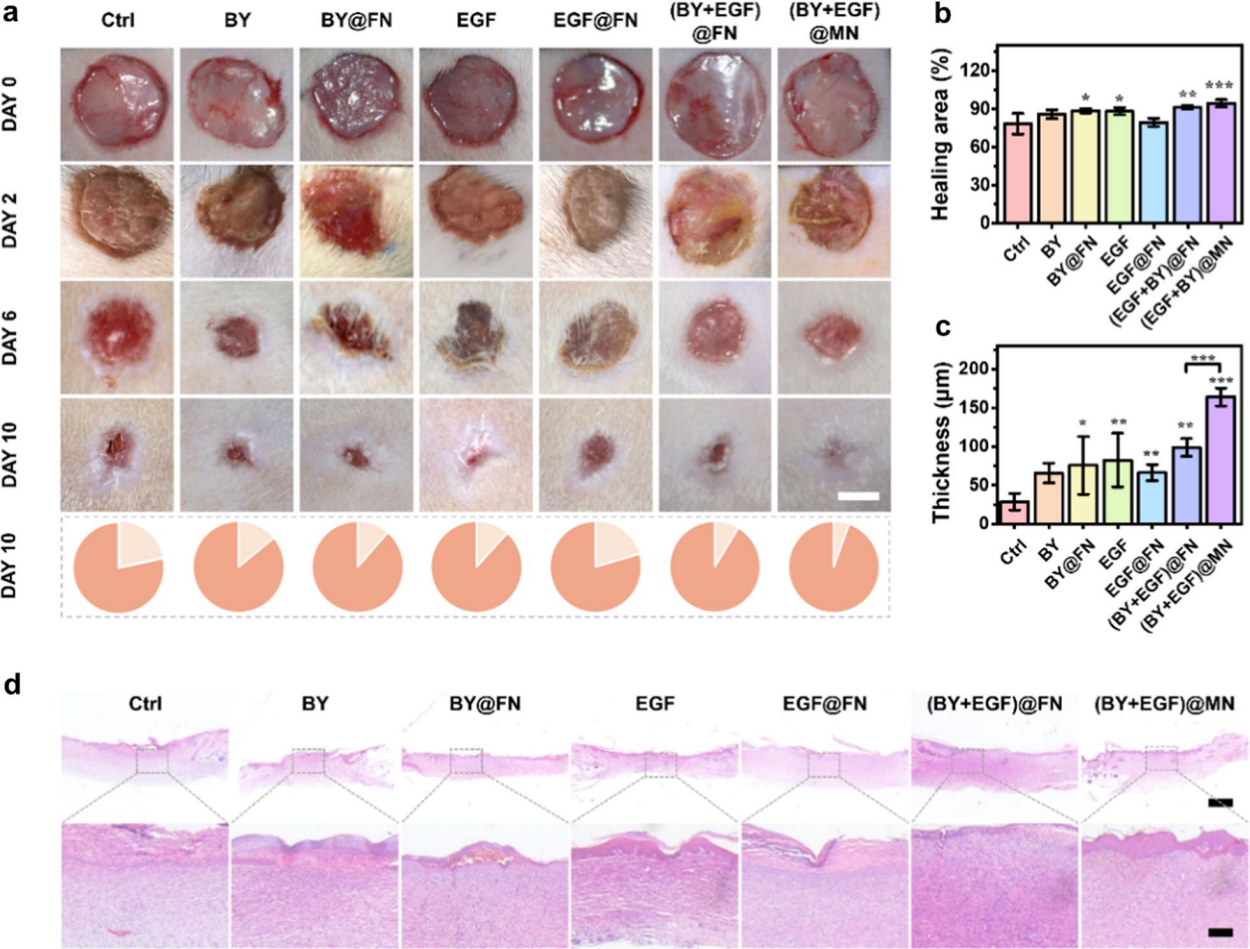
In vivo skin regeneration capacity of (BY + EGF)@MNs in a cutaneous wound model. **a** Optical photos of the skin wounds with different treatments for10 days. **b** Quantitative analysis of the healing area on day 10 (n = 5). **c** Quantitative analysis of epidermal thickness obtained from H&E staining on day 10 (n = 5). **d** H&E staining images in different groups on day 10. Scale bars are 5 mm in (**a**), 1 mm in (**d**, top), and 100 μm in (**d**, bottom)

The in vivo regenerative capacity of (BY + EGF)@MNs was further investigated using a full-thickness cutaneous defect model (Fig. 6a). As expected, the (BY + EGF)@MN group showed a best wound closure among all groups (Fig. 6b). The regenerated skin tissues were harvested on day 10 for histological analysis. H&E staining revealed that the (BY + EGF)@MN group had a highest epidermal thickness and complete structure as compared to other groups (Fig. 6c, d). It was assumed that the EGF and BY could be released with the gradually-degraded (BY + EGF)@MNs, and the drug molecules could reach the deep dermis in combination with microneedle administration. Therefore, the wound healing ability of (BY + EGF)@MNs was significant higher than that of (BY + EGF)@FNs.

**Fig. 7:**
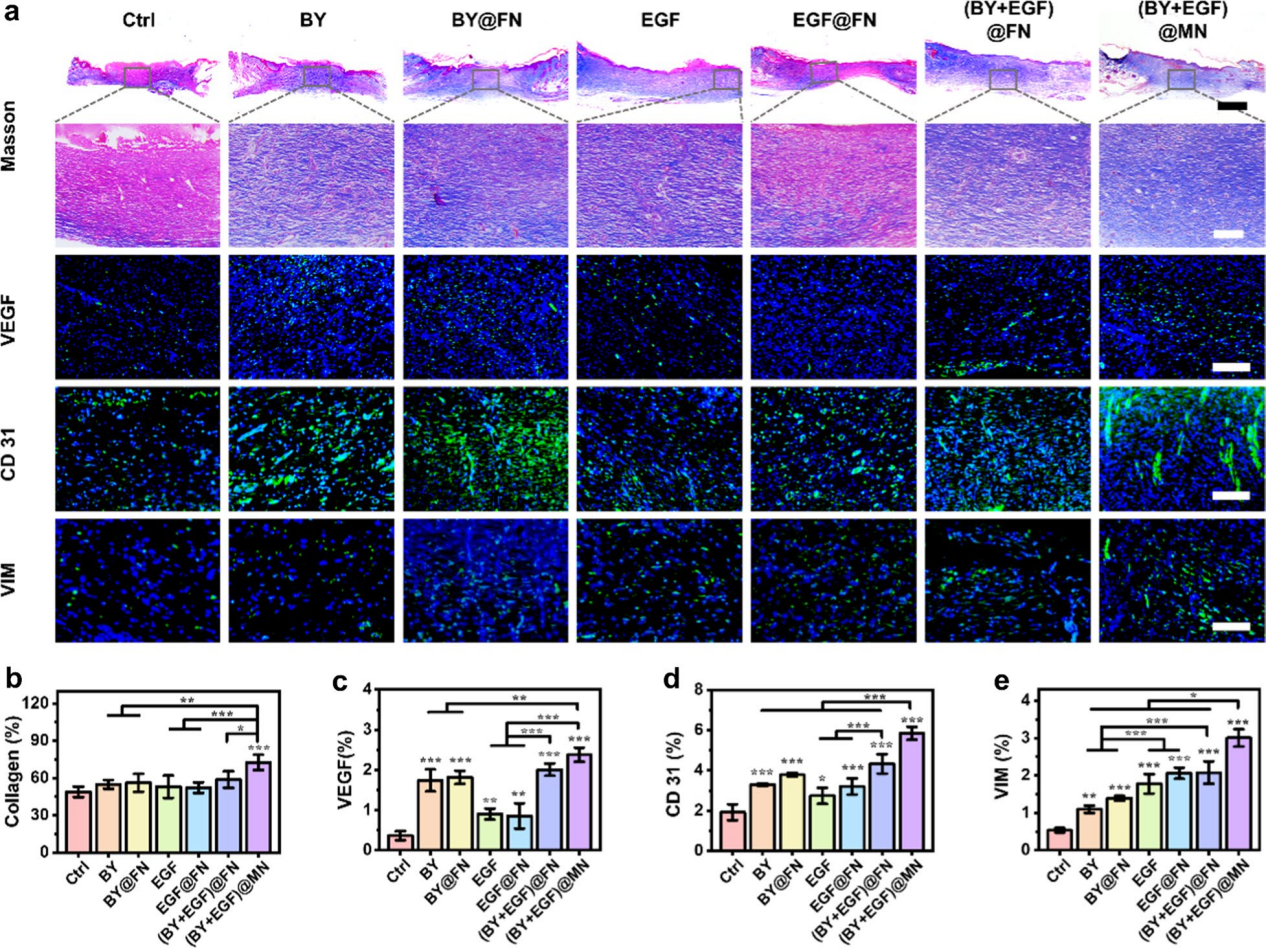
Histology analysis of the stimulatory effect of (BY + EGF)@MNs on skin wound healing. **a** Masson, immunofluorescence staining (including VEGF, CD31, VIM) of images on day 10. The VEGF, CD31, VIM are indicated in green in the corresponding images. Scale bars are 2 mm, 200 μm, 400 μm, 50 μm, and 100 μm from top to bottom in (**a**). **b**–**e** Quantitative analysis of the collagen deposition, VEGF, CD31, and vimentin synthesis (VIM) (n = 5)

Furthermore, Masson staining and immunofluorescence staining of vascular endothelial growth factor (VEGF), platelet endothelial cell adhesion molecule-1 (CD31), α-smooth muscle actin (α-SMA) and vimentin (VIM) were performed to evaluate the stimulatory effect of (BY + EGF)@MNs on collagen deposition and angiogenesis in the skin tissues (Fig. 7 and Additional file 1: Fig. S12). We observed that the collagen deposition was remarkably enhanced by the (BY + EGF)@MNs (73.24 ± 4.68%) in compassion to the BY (54.89 ± 3.20%), BY@FN (56.17 ± 6.46%), EGF (53.02 ± 8.04%), EGF@FN (52.85 ± 3.00%), and (BY + EGF)@FN (58.71 ± 5.97%) groups (Fig. 7a, b). VEGF, CD31, and α-SMA are known as markers for blood vessels, and the neovascularization are important for providing supply of nutrients and oxygen to the newly-regenerated tissues during wound healing. Our quantitative data showed that the relative VEGF expressions in the BY, BY@FN, (BY + EGF)@FN, (BY + EGF)@MN, EGF, and EGF@FN groups were 1.74 ± 0.24%, 1.81 ± 0.14%, 2.01 ± 0.14%, 2.38 ± 0.16%, 0.90 ± 0.12%, and 0.86 ± 0.28%, respectively (Fig. 7c). The relative CD31 expressions in the BY, BY@FN, (BY + EGF)@FN, (BY + EGF)@MN, EGF, and EGF@FN groups were 3.29 ± 0.06%, 3.78 ± 0.09%, 4.33 ± 0.43%, 5.85 ± 0.29%, 2.74 ± 0.36%, and 3.20 ± 0.36%, respectively (Fig. 7d). Similarly, the relative α-SMA expressions in the BY, BY@FN, (BY + EGF)@FN, (BY + EGF)@MN, EGF, and EGF@FN groups were 2.59 ± 0.79%, 2.60 ± 0.70%, 2.85 ± 0.72%, 4.61 ± 0.63%, 1.33 ± 0.56%, and 1.15 ± 0.37%, respectively (Additional file 1: Fig. S12). All these data indicated that the BY-containing groups displayed a higher vascular density than other groups, further confirming the remarkable angiogenic ability of BY that could be released from the (BY + EGF)@MNs.

Additionally, VIM immunohistochemical staining was used for evaluating cytoskeleton integrity in the newly-regenerated tissues [30, 31]. It was found that the VIM expressions in the EGF (1.78 ± 0.23%), EGF@FN (2.06 ± 0.13%), (BY + EGF)@FN (2.08 ± 0.27%), and (BY + EGF)@MN (3.00 ± 0.21%) groups were significantly higher than those in BY(1.10 ± 0.89%) and BY@FN (1.39 ± 0.06%) groups (Fig. 7e). Collectively, these results demonstrated that (BY + EGF)@MNs could significantly accelerate the wound healing in vivo by enhancing neovascularization, fibroblast density, and collagen deposition.

## Conclusion

In summary, we presented Yunnan Baiyao-loaded multifunctional microneedle patches (i.e. (BY + EGF)@MNs) for hemorrhagic wound healing. Owing to BY-loaded BSP base for rapid hemostasis and EGF-loaded GelMA tips for enhanced tissue regeneration, the (BY + EGF)@MNs not only exhibited significant pro-coagulability in vitro and hemostatic function in vivo, but also accelerated the neovascularization and collagen deposition during in vivo wound healing process. Such (BY + EGF)@MNs are believe to be promising candidates for rapid hemostasis and diverse tissue repairing applications.

## Experimental section

*Materials and animals* Gelatin, methacrylic anhydride (MA), and 2-hydroxy-2-methylpropylenone (HMPP) were obtained from Shanghai Aladin Co., Ltd., China. Bletilla polysaccharide (BSP) was purchased from Bai chuan Bio-technology Co., Ltd, China. Carbomer 940 was acquired from Beijing Solaibao Technology Co., Ltd, China. rRtEGF was bought from Shanghai PrimeGene Bio-tech Co., Ltd, China. Yunnan Baiyao (BY) was purchased from Yunnan Baiyao Group. Gelatin methacrylate (GelMA) was self-synthesized in our laboratory. Deionized water (DIW, 18.25 MΩ·cm^− 1^, Millipore) was used through all experiments. Male Sprague-Dawley rats weighted 125–150 g were provided by Vital River Laboratory Animal Technology Co., Ltd. Any animal experimental protocols were approved by the Animal Investigation Ethics Committee of Wenzhou Institute of University of Chinese Academy of Sciences (No. WIUCAS22092203).

*Synthesis of GelMA* GelMA was synthesized in our laboratory as we previously published [32]. Typically, the gelatin (10%, w/v) and Na_2_CO_3_ (5%, w/v) were dissolved in deionized water (DIW) and stirred for 2 h. 5 mL of methacrylic anhydride was dropwise added into 200 mL of gelatin solution within 30 min and stirred for another 2 h. During the reaction process, the pH value of the mixed solution was adjusted by NaOH solution (1 M) for maintaining 8–9. The resultant solution was dialyzed against DIW before lyophilization to obtain GelMA. The degree of substitution was examined from ^1^ H NMR spectra (QUANTUM-I-400 MHz, Q.One Instruments, China).

*Fabrication of (BY + EGF) @MNs* GelMA (20%, g/mL), EGF (0.002%, g/mL) and HMPP (1%, g/mL) were dissolved in the DIW to obtain the EGF-GelMA pre-gels, which 100 µL EGF-GelMA pre-gels were used to fill the MN tip cavity in the polydimethylsiloxane (PDMS) mold using a vacuum pump. Excess solution was removed (≥ 50 µL) and then cured under UV light (365 nm, 10 W, 10 s). BSP (10% w/v), Carbomer 940 (0.6%, g/mL) and BY (0.8%, g/mL) were dissolved in the DIW to obtain the BY-BSP pre-gels, which about 100 µL BY-BSP pre-gels were used to fill MN base cavity in the PDMS mold and subsequently frozen at –80 ℃. The (BY + EGF)@MNs were finally obtained after lyophilization.

*Characterization* The bright-field and fluorescence micrographs of (BY + EGF)@MNs were observed using a stereomicroscope (Olympus BX51, Tokyo, Japan). The microscopic structures of (BY + EGF)@MNs were recorded by a scanning electron microscope (SU8010, Hitachi, Japan). The compression and tensile tests of (BY + EGF)@MNs were performed on a universal mechanical testing machine (5944, Instron, USA).

*Mechanical characterizations* The puncture strength of MN tips was measured by a compression test. The MN patches with different GelMA concentrations were laid on a platform, where the MN tips pointed to the descending pressure sensor. The sensor gradually moved to the tips at 2 mm/min, and the force began to be recorded as 0.01 N when touching the MN tips. The force-displacement curves were recorded, and the force at 500 nm for different MN tips were calculated. In addition, the (EGF + BY)@MNs were inserted into the rat skin and liver tissues, which were stained with H&E to evaluate the puncture intensity.

*Tissue adhesion tests* The adhesion strength between the skin tissues and MN bases was verified by a tensile test. The pig skin tissues were firstly fixed to the horizontal stage. MN bases with gradient Carbomer contents were contacted with the skin tissues under a pre-pressure of 5 N for 1 min. The upside pressure sensor attached to the MN bases was stretched upwards at a speed of 20 mm/min. The stretching process was terminated manually when the MN bases were separated from the skin tissues. The maximum tensile force could be obtained from the force-displacement curves as an index to evaluate the adhesion ability of the MN bases.

*BSP dissolution and BY release* The release kinetics of BSP and BY were assessed using a UV absorption spectroscope (obtained by Agilen CARY5000, USA). BSP and BY had the highest absorbance at 280 nm and 260 nm. The BSP patches with different Carbomer concentrations were placed in 5 mL of PBS (PH 5.6). Subsequently, 0.5 mL of PBS leaching solution was taken out every 1 min, and then an equal amount of identical PBS was added immediately. By measuring the absorbance at 280 nm, the BSP dissolution rate could be calculated based on the standard curve of BSP solution. Similarly, the release kinetics curves of BY were obtained by treating BY-containing BSP film (BY@FN) in PBS solution under different pH conditions (i.e., pH = 5.6, 6.5, and 7.4) with the same way.

*GelMA Degradation and drug release* The degradation rate of GelMA MN tips was evaluated by recording their weight loss. We prepared cube-shaped GelMA hydrogel samples (size: 16 mm× 16 mm× 1 mm) to avoid interference of BSP-based MN bases. All samples were immersed in 1 ml of PBS with collagenase II (2.6 U/mL), kept shaking at 37 °C. The PBS solution was replaced at regular intervals after centrifugation, and then 1 mL of fresh PBS was supplemented. The MNs were dried completely at 60 °C and their dry weights were recorded at designed time pints. The degradation curve was plotted based on the weight loss. For drug release test, BSA was used as a drug model and be incorporated into the same cube-shaped GelMA hydrogel to avoid interference of BSP-based MN bases. With the same manner as above, the absorbance of BSA released in the leaching solution were detected using UV spectroscopy at each time point. The BSA release profiles were obtained under different pH conditions.

*RBC and platelet adhesion assays* Gauze (clinical cotton gauze), BSP@FN (BSP flat patches, fabricated by 100 µL pre-gels with 10% BSP and 0.6% carbomer (w/v) after freeze-drying), BY@FN (BY-loading BSP flat patches, fabricated by 100 µL BY-BSP pre-gels (g/mL) containing 0.8% BY, 10% BSP and 0.6% carbomer), and (EGF + BY)@MNs (MN patches MN patches, fabricated by 100 µL BY-BSP pre-gels (g/mL) containing 0.8% BY, 10% BSP and 0.6% carbomer and 50 µL EGF-GelMA pre-gels (g/mL) containing 0.002% EGF and 20% GelMA ) were used as experimental groups. The adhesion experiments were carried out as published [33]. RBC suspension and platelet-rich plasma (PRP) were separated from the citrated whole blood (CWB) by centrifugation (1500 rpm, 10 min).

For RBC adhesion assay, the RBCs suspensions (100 µL) were paved on the MN patch surface. After 1 min, they were washed to remove non-adherent RBCs by using PBS solution, and then soaked in DIW (4 mL) to release hemoglobin. After 2 h, the supernatant (100 µL) was pipetted, and the OD value was measured at 540 nm (OD _experimental_). The OD value of DIW (4 mL) with RBCs suspension (100 µL) and PBS (100 µL) was measured as positive value (OD _positive_) and negative value (OD _negative_), respectively. The rate of adhered RBCs was calculated by the following Eq. (1):

1 $${\text{RBC}_{{\text{adhesion}}}}(\% ) = \frac{{{\text{(OD}_{{\text{experimental}}}}-{\text{OD}_{{\text{negative}}}}{\text{)}}}}{{{\text{(OD}_{{\text{positive}}}}-{\text{OD}_{{\text{negative}}}}{\text{)}}}} \times 100\%$$

For platelet adhesion assay,100 µL of PRP was paved onto the sample surfaces for 1 min. Non-adherent platelets were removed with PBS, and then the samples were soaked in 2 mL of Triton X-100 solution to free the lactate dehydrogenase (LDH). The LDH contents in the supernatants were determined using a LDH kit (Solarbio, China). The OD_450 nm_ value of the Triton X-100 solution containing 100 µL of PBS and PRP was used as negative value (OD _negative_) and positive value (OD _positive_), respectively. The ratio of adhered platelets was calculated by the following Eq. (2):

2 $${\text{Platelet}_{{\text{adhesion}}}}(\% ) = \frac{{{\text{(OD}_{{\text{experimental}}}}-{\text{OD}_{{\text{negative}}}}{\text{)}}}}{{{\text{(OD}_{{\text{positive}}}}-{\text{OD}_{{\text{negative}}}}{\text{)}}}} \times 100\%$$

In addition, the adhered RBCs and platelets were observed by SEM. The SEM samples were prepared by being incubated with 100 µL RBC or PRP for 1 min, washed with PBS, fixed in 3% glutaraldehyde for 12 h, dehydrated in gradient ethanol solutions, dried with a supercritical dryer, and then sprayed with gold by a high-vacuum ion sputtering machine (Leica, EM ACE600).

*Coagulation assessments* The blood clotting test of (EGF + BY)@MNs was performed according to previous studies [34]. Normal (without treatment), BY (BY powers, 1 mg), BY@FN (BY-loading BSP flat patches, fabricated by 100 µL of BY-BSP pre-gels containing 0.8% BY, 10% BSP and 0.6% carbomer), and (EGF + BY)@MNs (MN patches with a BY-loading BSP base and EGF-loading GelMA tips, fabricated by 100 µL of BY-BSP pre-gels and 50 µL of EGF-GelMA pre-gels (g/mL) containing 0.002% EGF and 20% GelMA) were ground and mixed with the 1 mL of serum from CWB. The clotting times of different groups were recorded at 37 ℃ by the PT/APTT assay reagent kits (Beijing ZONCI Technology Development Co., Ltd, China).

*Platelet activation assessments* P-selectin (CD 62p) immunofluorescence staining was performed to evaluate the activation effect of (EGF + BY)@MNs. The samples preparation in different groups were as same as RBC and platelet adhesion assays, which were fabricated with co-incubated with PRP for 10 min, horizontally placed on glass slides, and then fixed in glutaraldehyde for 12 h. The staining was performed sequentially by CD 62p monoclonal antibody, and then examined using a fluorescence microscope.

*Hemocompatibility assessments* Hemocompatibility of (EGF + BY)@MNs was assessed by observation and quantification of hemoglobin released from the RBCs incubated with different samples. RBC suspensions were acquired from CWB by centrifugation. 100 µL of RBCs suspension was mixed with 900 µL of leaching solution for different samples, which was centrifuged after incubation for 1 h. This hemolysis process was photographed using a digital camera. The OD values in different experimental groups were recorded as OD _experimental_ at 550 nm. 100 µL RBCs suspension was dropped into DIW and used as positive control (OD _positive_), while PBS was used as negative control (OD _negative_). The hemolysis rate was obtained by following Eq. (3):

3 $${\text{Hemolysis rate}_{{\text{adhesion}}}}(\% ) = \frac{{{\text{(OD}_{{\text{experimental}}}}-{\text{OD}_{{\text{negative}}}}{\text{)}}}}{{{\text{(OD}_{{\text{positive}}}}-{\text{OD}_{{\text{negative}}}}{\text{)}}}} \times 100\%$$

*Cytocompatibility assessments* 0 and 1 mg/ml of leaching medium of (BY + EGF)@MNs were used as control and experimental group, respectively. NH3T3 cells were added in a 96-well plate (800/well) and cultivated by different solutions. On days 1, 2, and 3, the OD value was detected at 450 nm after incubating with CCK-8 solution (10% v/v, Beyotime Biotechnology) for 4 h. The NH3T3 cells were stained with a live/dead staining kit for morphology observation.

*Hemostasis and liver wound healing in vivo* In all animal experiments, SD rats were divided into 7 groups: Ctrl (without treatment), BY (treated with BY powders, 1 mg), BY@FN (treated with BY-loading BSP flat patches, fabricated by 100 µL of BY-BSP pre-gels containing 0.8% BY, 10% BSP and 0.6% carbomer), EGF (treated with 10 µL of 0.01% g/mL EGF solution), EGF@FN (treated with EGF-loading GelMA flat patches, fabricated by 50 µL of EGF-GelMA pre-gels containing 0.002% EGF and 20% GelMA), (EGF + BY)@FN (treated with EGF-loading GelMA and BY-loading BSP flat patches, fabricated by 50 µL of EGF-GelMA pre-gels and 100 µL of BY-BSP pre-gels), (EGF + BY)@MNs (treated with MN patches with EGF-loading GelMA tips and a BY-loading BSP base, fabricated by 50 µL of EGF-GelMA pre-gels and 100 µL of BY-BSP pre-gels). The drug or patch in each group were administered only once on day 0. Additionally, before traumatic procedures, all animals were anesthetized by injecting atropine sulfate (0.01 ml/100 g, 0.4 mg/mL, Shanghai full woo Biotechnology Co., Ltd., China) into muscular and Zoletil®50 (0.15 ml/100 g, 50 mg/mL, Virbac Co., Ltd., France.) into abdominal cavity.

The in vivo hemostatic and liver regenerative capacities of the (EGF + BY)@MNs were evaluated using a liver puncture hemorrhage model in SD rats. The rat abdomen was opened after anesthetization. The livers were laid onto a pre-weighed sterile filter paper. Next, a “cross” wound with a length of 5 mm and a depth about 3 mm was made for hepatic hemorrhage. The liver wounds were covered with different samples, and the hemostasis process was recorded. The hemostasis time was measured by a timer. The blood loss was determined by weighting the filter papers. The live tissues in different groups were harvested after 28 days. H&E staining (Solarbio, Beijing), PAS staining (Beyotime, Shanghai) and IL-6 immunohistochemical staining (Servicebio, Wuhan) were conducted for histological analysis.

*In vivo tissue repair evaluation* A full-thickness cutaneous wound mode was established in rats to demonstrate the tissue repair effect of (EGF + BY)@MNs. The cutaneous tissues on the back of anesthetized rats were excised to form a circle with a radius of 5 mm and treated with different ways in various groups. The wounds were divided into 7 groups: Ctrl (without treatment), BY (treated with BY powders, 1 mg), BY@FN (treated with BY-loading BSP flat patches, fabricated by 100 µL of BY-BSP pre-gels (g/mL) containing 0.8% BY, 10% BSP and 0.6% carbomer), EGF (treated with 10 µL of 0.01% EGF solution), EGF@FN (treated with EGF-loading GelMA flat patches, fabricated by 50 µL of EGF-GelMA pre-gels containing 0.002% EGF and 20% GelMA), (EGF + BY)@FN (treated with EGF-loading GelMA and BY-loading BSP flat patches, fabricated by 50 µL of EGF-GelMA pre-gels and 100 µL of BY-BSP pre-gels), (EGF + BY)@MNs ( treated with MN patches with EGF-loading GelMA tips and a BY-loading BSP base, fabricated by 50 µL of EGF-GelMA pre-gels and 100 µL of BY-BSP pre-gels). Subsequently, all wounds were covered with medical breathable tapes to avoid unwanted scratches. For tracking the wound healing process, the wounds were photographed on days 0, 2, 6, and 10. The wound dressings were not renewed at the designed intervals, which could be beneficial for monitoring the in vivo degradation of (EGF + BY)@MNs. Regenerated skin tissues were collected from the sacrificed rats on day 10. Microanatomy analysis was conducted by typical H&E/Masson staining, and immunofluorescence staining of CD31, VEGF, α-SMA and VIM.

*Statistical analysis* All quantitative analysis were conducted as means with standard deviations (n ≥ 3). Differences of two groups and multiple groups were analyzed by unpaired Student’s t-tests and one-way analysis-of-variance (ANOVA), respectively. Significant differences were considered when *p < 0.05, **p < 0.01, and ***p < 0.001, and ‘ns’ indicated no significant difference.

## Supplementary Information

**Additional file 1:** **Figure S1. a** ^1^H NMR spectraof GelMA and gelatin in D_2_O. **Figure S2.** **a** Optical images of@MNs. **b**, **c** Cross-sectional SEM images of theMN tips. Scale bars are 2 mm in, 200 µm in, and 50 µm in. **Figure S3.** **a**, **b** Digital photosand H&E staining images of the puncture of the@MNs on skinandlivertissues in rats. Scale bars are 2mm, 2 mm, and 200 µm from left toright in. **Figure S4.** UV spectra of BSP,BY, and Carbomer hydrogels. **Figure S5.** The pro-activatingplatelet ability. **a** SEM images of platelets activated by the@MNs. **b** Immunofluorescence staining of CD62p indicating the activation of platelets bythe@MNs. Scale bars are 50 µm, 25 µm, 10 µm, 2.5 µm from left to rightin, and 50 µm in. **Figure S6**. **a** Visualization images of activated partialthromboplastin timein different groups. **b** Quantitative analysis ofAPPT. **Figure S7**. Respective photographs and corresponding quantitativeanalysis of hemocompatibility for the **a** EGF, **b** BY and **c** @MNs. **Figure S8.** Cytocompatibility ofthe@MNs. **a** CCK-8 assay of the NIH3T3 cells cultured with the@MNsfor 3 days. **b **Live/dead staining images ondays 1, 2, and 3. The scale bar is 100 µm in. **Figure S9.** **a** Representativeoptical images of the scratch assay of the NIH3T3 cells cultured in gradientEGF solutions. **b** Quantification of closure rates in the scratch assay. The scale bar is100 µm in. **Figure S10.** Quantitative analysisof **a** AST and **b** ALT on day 28. **Figure S11. a** Representative immunofluorescent stainingimages and **b** semi-quantitative analysis of α-SMA. **Figure S12. a** Representative immunofluorescent stainingimages and **b** semi-quantitative analysis of α-SMA. The α-SMA isindicated in green. Scale bar are 200 µm in.  

## Data Availability

All data analyzed during this study are included in this published article and its supplementary information files.
