# Supplementary material for: Yunnan Baiyao-loaded multifunctional microneedle patches for rapid hemostasis and cutaneous wound healing
Source: J Nanobiotechnology. 2023 Jun 6;21:178. doi: 10.1186/s12951-023-01936-w (PMC10242798; doi:10.1186/s12951-023-01936-w)
Supplement: Supplementary file 1 — Additional file 1: Figure S1. a 1H NMR spectraof GelMA and gelatin in D2O. Figure S2. a Optical images of@MNs. b, c Cross-sectional SEM images of theMN tips. Scale bars are 2 mm in, 200 µm in, and 50 µm in. Figure S3. a, b Digital photosand H&E staining images of the puncture of the@MNs on skinandlivertissues in rats. Scale bars are 2mm, 2 mm, and 200 µm from left toright in. Figure S4. UV spectra of BSP,BY, and Carbomer hydrogels. Figure S5. The pro-activatingplatelet ability. a SEM images of platelets activated by the@MNs. b Immunofluorescence staining of CD62p indicating the activation of platelets bythe@MNs. Scale bars are 50 µm, 25 µm, 10 µm, 2.5 µm from left to rightin, and 50 µm in. Figure S6. a Visualization images of activated partialthromboplastin timein different groups. b Quantitative analysis ofAPPT. Figure S7. Respective photographs and corresponding quantitativeanalysis of hemocompatibility for the a EGF, b BY and c @MNs. Figure S8. Cytocompatibility ofthe@MNs. a CCK-8 assay of the NIH3T3 cells cultured with the@MNsfor 3 days. b Live/dead staining images ondays 1, 2, and 3. The scale bar is 100 µm in. Figure S9. a Representativeoptical images of the scratch assay of the NIH3T3 cells cultured in gradientEGF solutions. b Quantification of closure rates in the scratch assay. The scale bar is100 µm in. Figure S10. Quantitative analysisof a AST and b ALT on day 28. Figure S11. a Representative immunofluorescent stainingimages and b semi-quantitative analysis of α-SMA. Figure S12. a Representative immunofluorescent stainingimages and b semi-quantitative analysis of α-SMA. The α-SMA isindicated in green. Scale bar are 200 µm in. [file 12951_2023_1936_MOESM1_ESM.docx]

**Yunnan Baiyao-Loaded Multifunctional Microneedle Patches for Rapid Hemostasis and Cutaneous Wound Healing**

*Jie Yang*^1,2^*, Xiaocheng Wang*^1,2^*, Dan Wu*^1,2^*, Kexin Yi*^1,2^*, Yuanjin Zhao^*^* ^1,2^

^1^ Department of Rheumatology and Immunology, Nanjing Drum Tower Hospital, School of Biological Science and Medical Engineering, Southeast University, Nanjing 210096, China

^2^ Oujiang Laboratory (Zhejiang Lab for Regenerative Medicine, Vision and Brain Health); Wenzhou Institute, University of Chinese Academy of Sciences, Wenzhou, Zhejiang 325001, China

* Corresponding author e-mail addresses: [yjzhao@seu.edu.cn](mailto:yjzhao@seu.edu.cn)


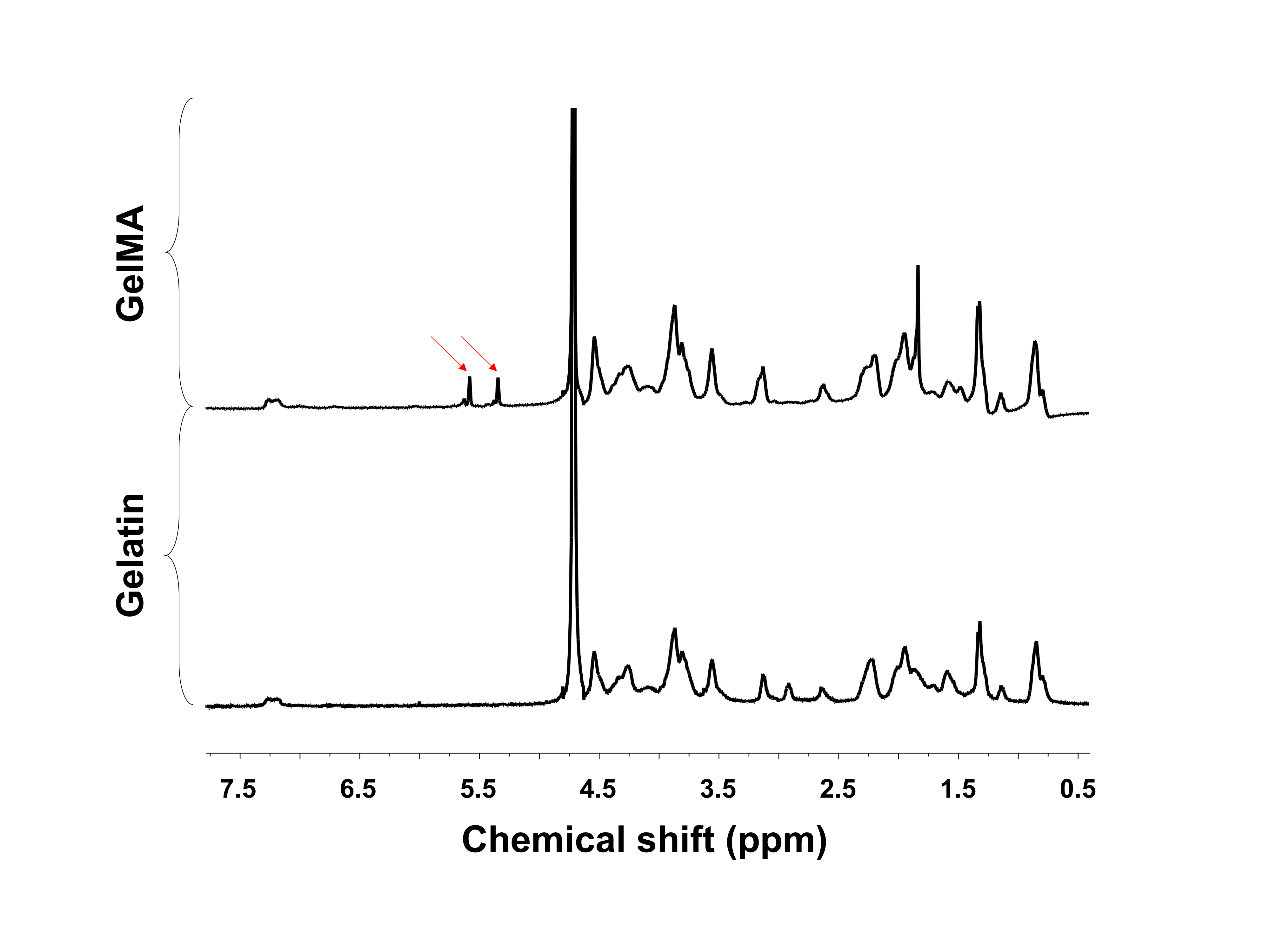


**Figure S1.** (a) ^1^H NMR spectra of GelMA and gelatin in D_2_O.


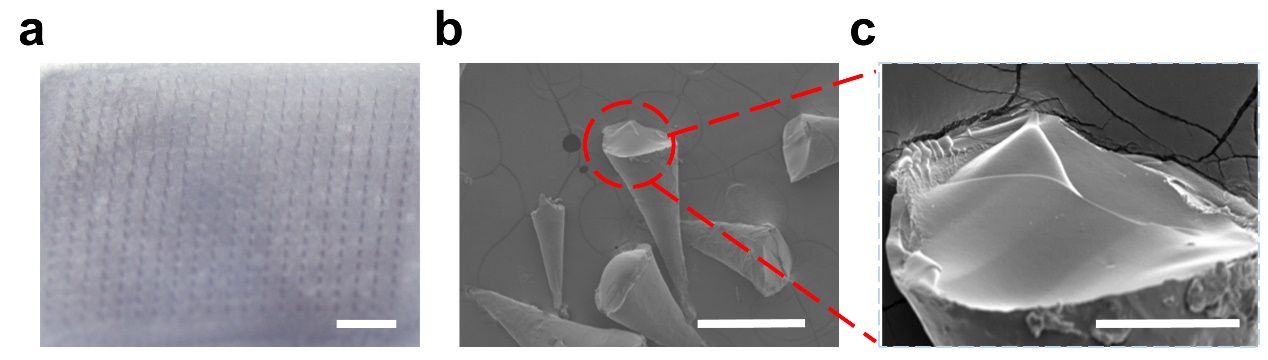


**Figure S2.** (a) Optical images of (BY+EGF)@MNs. (b-c) Cross-sectional SEM images of the MN tips. Scale bars are 2 mm in (a), 200 µm in (b), and 50 µm in (c).


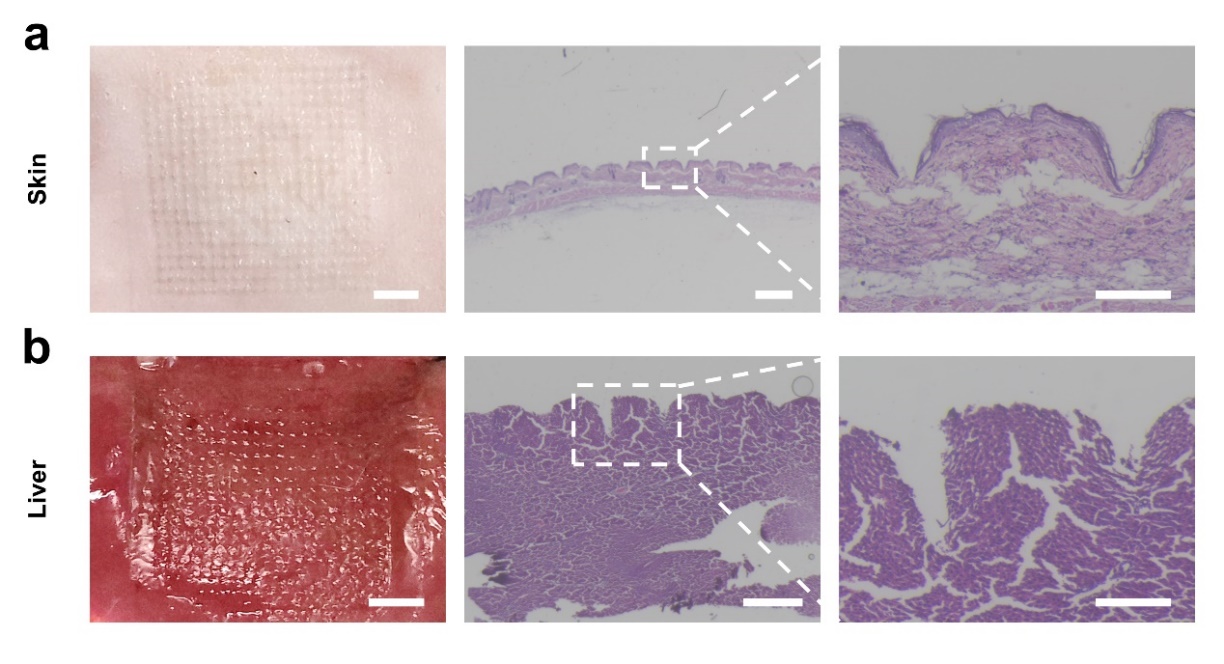


**Figure S3.** (a-b) Digital photos and H&E staining images of the puncture of the (BY+EGF)@MNs on skin (a) and liver (b) tissues in rats. Scale bars are 2mm, 2 mm, and 200 µm from left to right in (a, b).


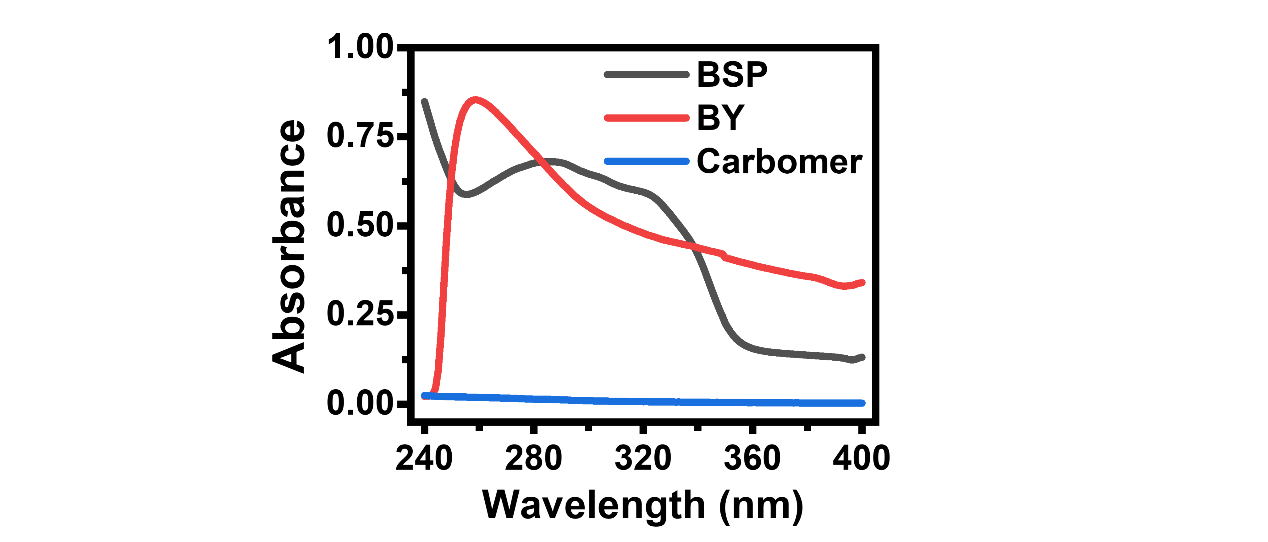


**Figure S**4. UV spectra of BSP, BY, and Carbomer hydrogels.


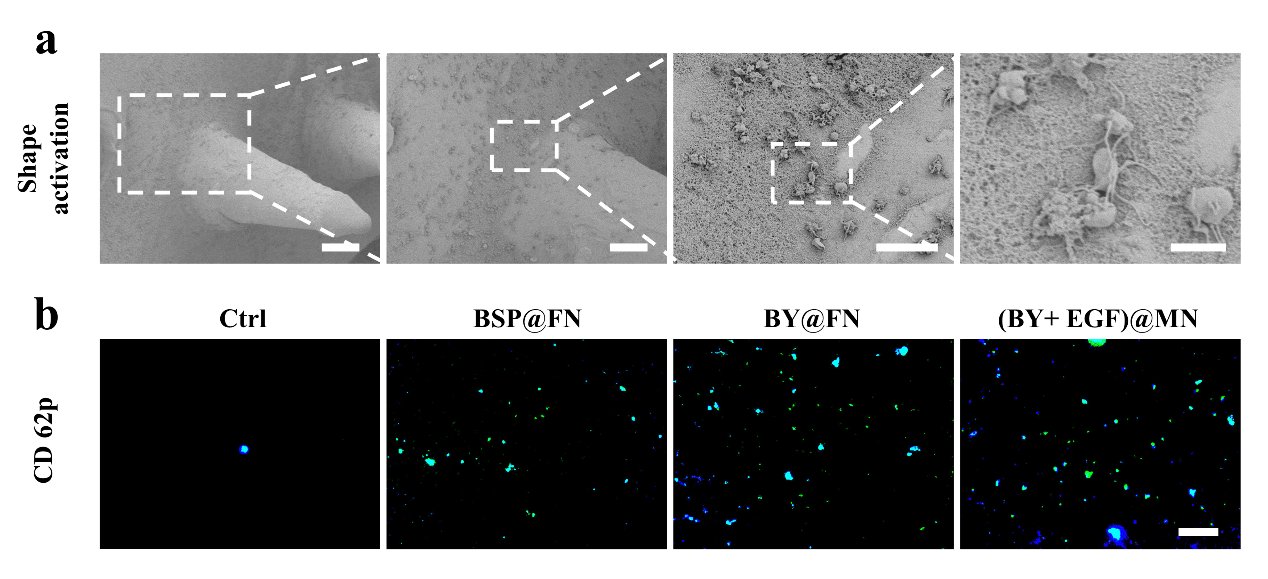


**Figure S5.** The pro-activating platelet ability. (a) SEM images of platelets activated by the (BY+EGF)@MNs. (b) Immunofluorescence staining of CD62p indicating the activation of platelets by the (BY+EGF)@MNs. Scale bars are 50 µm, 25 µm, 10 µm, 2.5 µm from left to right in (a), and 50 µm in (b).


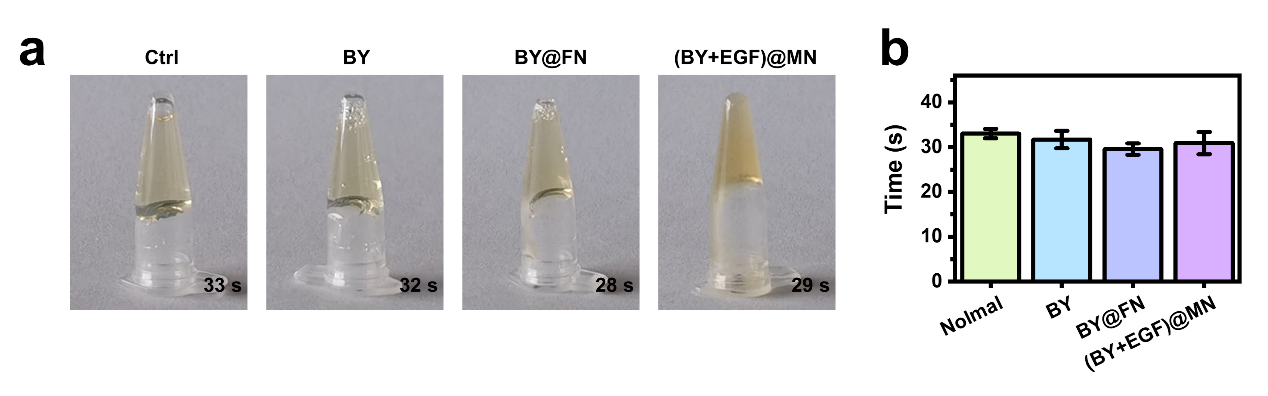


**Figure S6**. (a) Visualization images of activated partial thromboplastin time (APPT) in different groups. (b) Quantitative analysis of APPT (n=5).

**
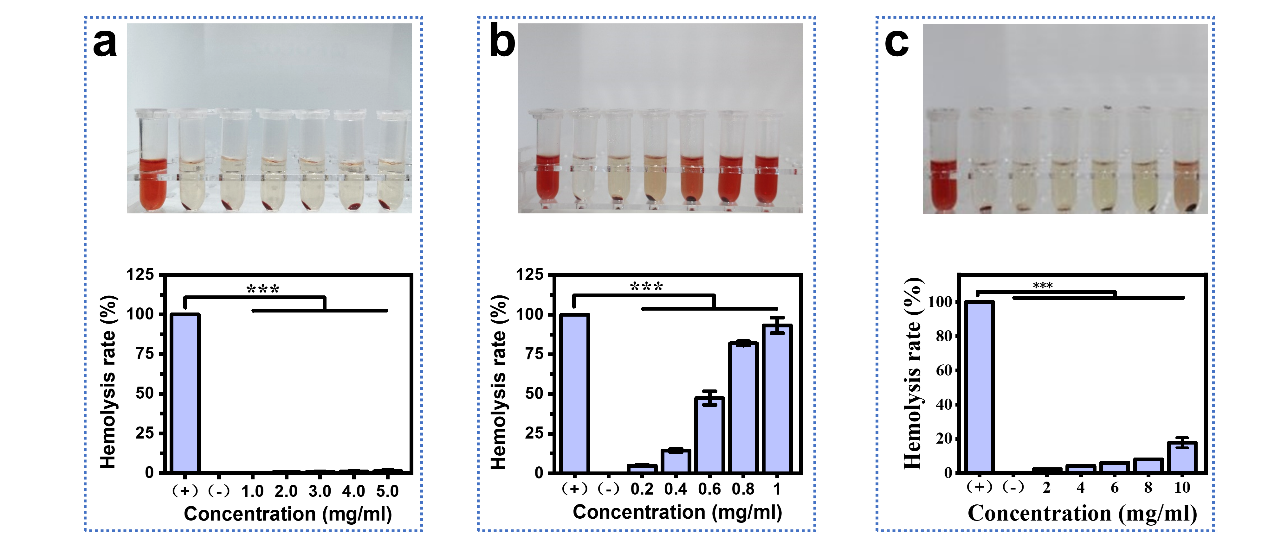
**

**Figure S7**. Respective photographs and corresponding quantitative analysis of hemocompatibility for the (a) EGF, (b) BY and (c) (BY+EGF)@MNs (n=3).


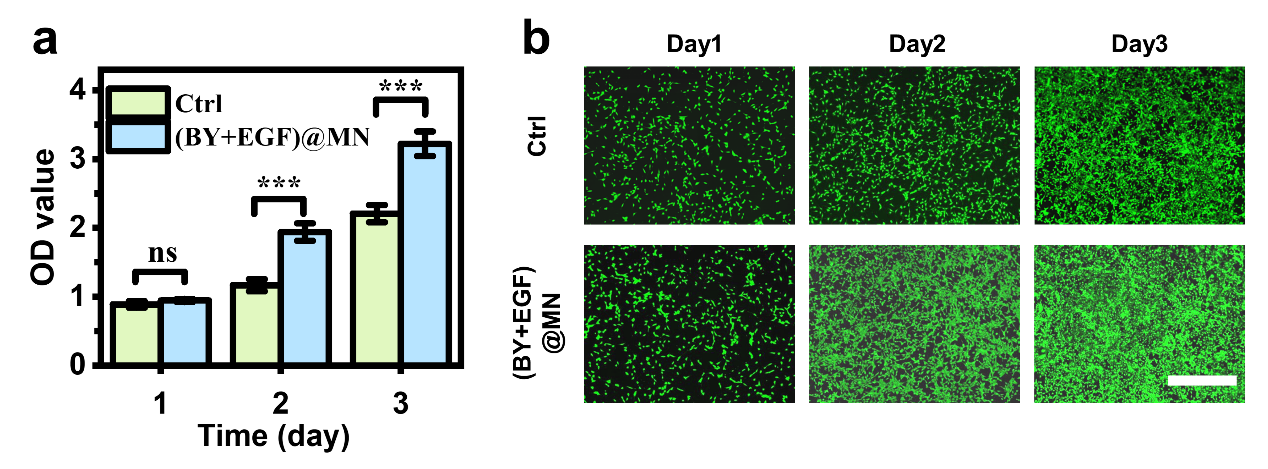


**Figure S8.** Cytocompatibility of the (BY+EGF)@MNs. (a) CCK-8 assay of the NIH3T3 cells cultured with the (BY+EGF)@MNs (1 mg/ml) for 3 days (n = 5). (b) Live/dead staining images on days 1, 2, and 3 (red: dead, green: live). The scale bar is 100 µm in (b).


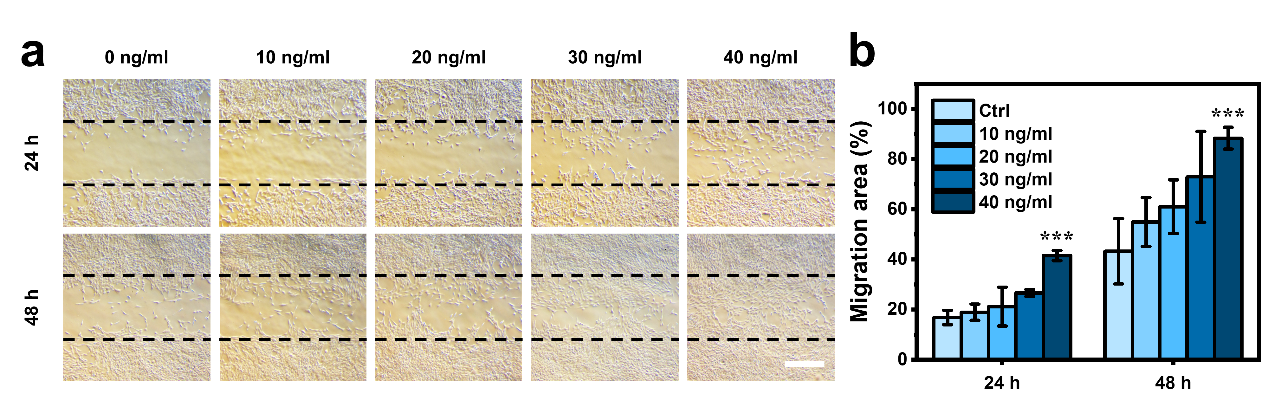


**Figure S9.** (a) Representative optical images of the scratch assay of the NIH3T3 cells cultured in gradient EGF solutions (Dotted lines indicate the initial scratch edges). (b) Quantification of closure rates in the scratch assay (n = 4). The scale bar is 100 µm in (a).


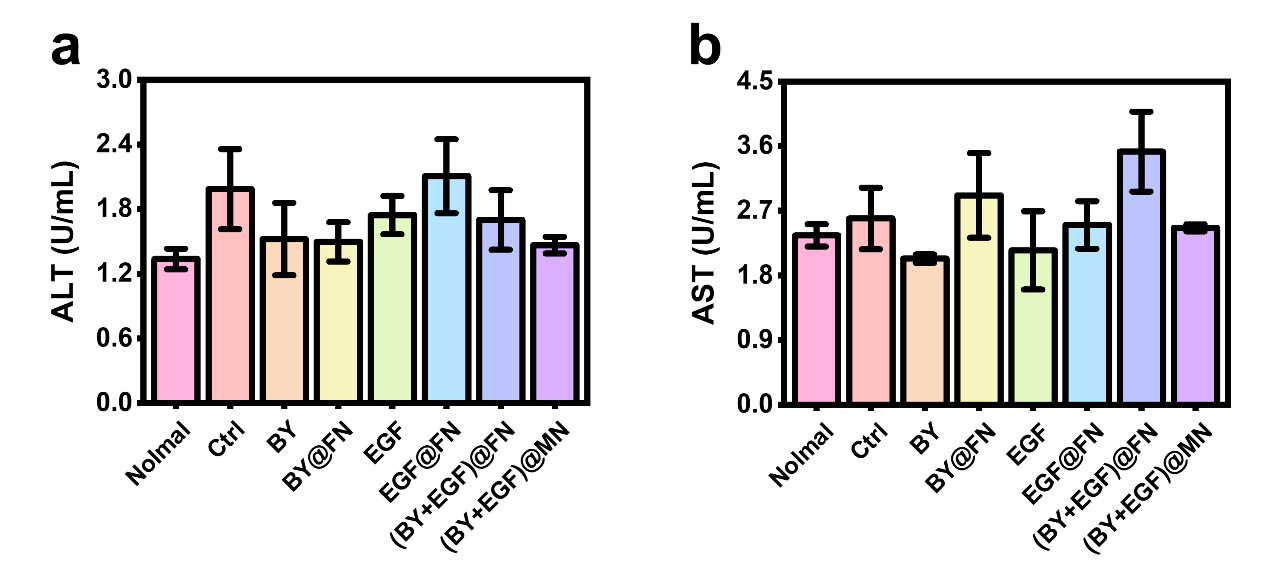


**Figure S10.** Quantitative analysis of (a) AST and (b) ALT on day 28 (n = 3).


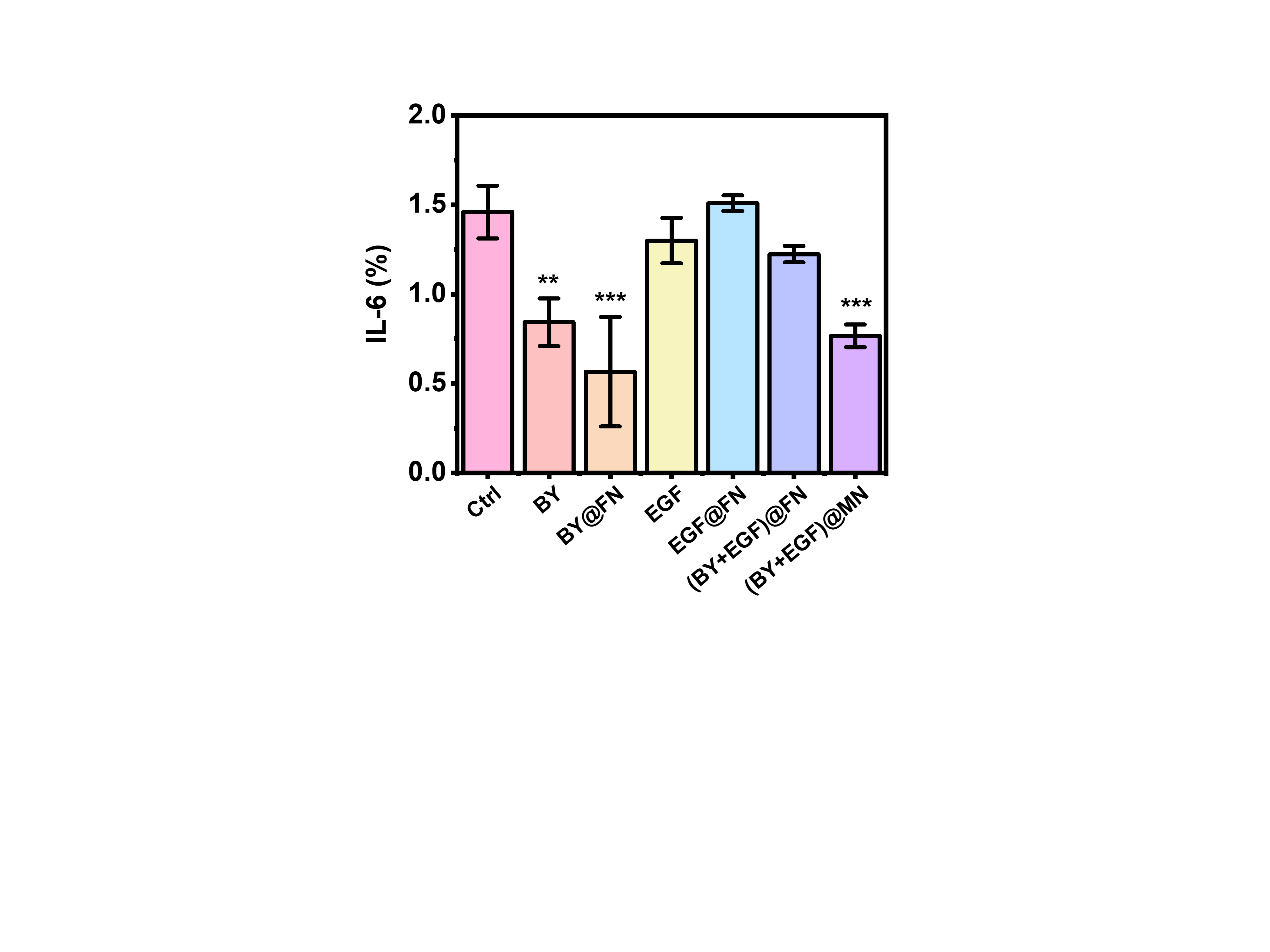


**Figure S11.** (a) Representative immunofluorescent staining images and (b) semi-quantitative analysis of α-SMA (n = 5).


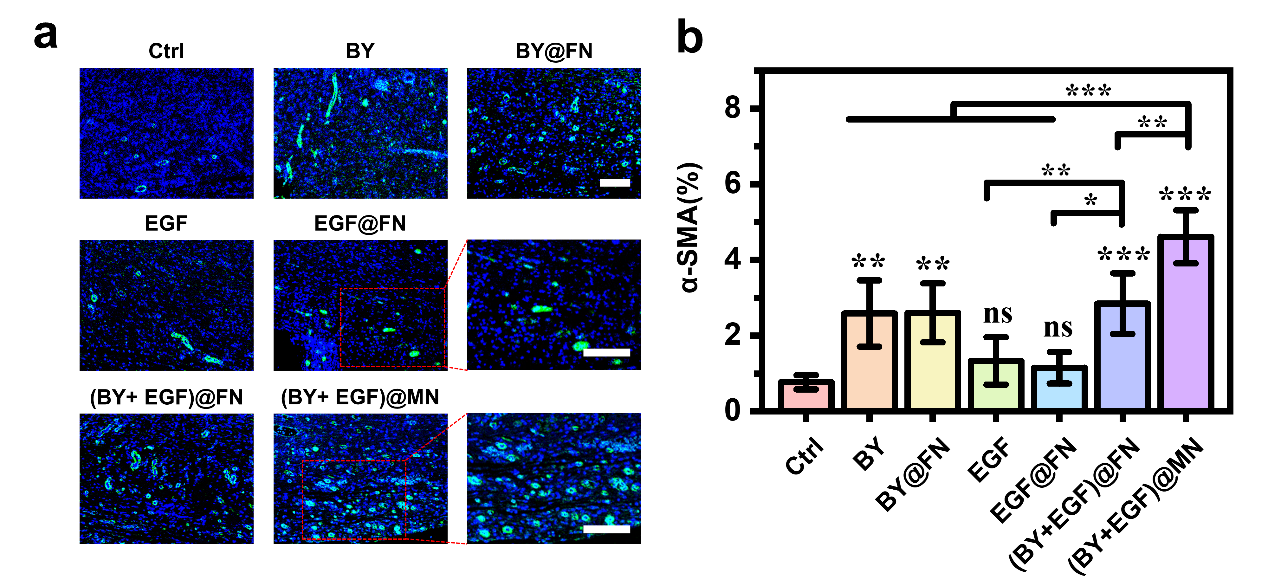


**Figure S12.** (a) Representative immunofluorescent staining images and (b) semi-quantitative analysis of α-SMA (n = 5). The α-SMA is indicated in green. Scale bar are 200 µm in (a).
